# Photothermal-triggered NO-releasing nanofiber membrane mitigates intervertebral disc degeneration via inflammation inhibition and matrix stabilization

**DOI:** 10.1016/j.mtbio.2025.102287

**Published:** 2025-09-04

**Authors:** Guanfeng Huang, Jiajun Xie, Jialan Chen, Jiangminghao Zhao, Pinkai Wang, Jian Zhang, Peichuan Xu, Yang Li, Xiaolong Chen, Xinxin Miao, Wei Xiong, Xigao Cheng

**Affiliations:** a Department of Orthopedics, The Second Affiliated Hospital, Jiangxi Medical College, Nanchang University, Nanchang, Jiangxi, 330006, China; b Jiangxi Provincial Key Laboratory of Spine and Spinal Cord Disease, Nanchang, Jiangxi, 330006, China; c Institute of Minimally Invasive Orthopedics, Nanchang University, Nanchang, Jiangxi, 330006, China; d Division of Orthopaedic Surgery, Department of Orthopaedics, Nanfang Hospital, Southern Medical University, Guangzhou, Guangdong, 510515, China

**Keywords:** Nanofibrous membrane, Nitric oxide gas therapy, Mild photothermal therapy, Annulus fibrosus injury

## Abstract

Intervertebral disc–related low back pain represents a major source of chronic pain. Due to the inflammatory microenvironment and impaired extracellular matrix (ECM) synthesis in intervertebral disc degeneration (IVDD), annulus fibrosus (AF) injuries have limited healing capacity. Effective AF repair thus requires modulation of the inflammatory state, promotion of ECM deposition, and enhancement of cellular migration. In this study, we developed a multifunctional photothermal nanofibrous membrane system by electrospinning biocompatible chitosan (CS) and polyvinyl alcohol (PVA) integrated with the photosensitizer polyaniline (PANI) and the nitric oxide (NO) donor S-nitrosoglutathione (GSNO). The system enables mild photothermal therapy (MPTT)–NO synergy via NIR-triggered photothermal heating and controlled NO release. Under NIR irradiation, the generated mild heat induces AF cells to upregulate heat shock proteins (HSPs), thereby enhancing tissue repair. Furthermore, leveraging the thermosensitive release of NO from GSNO, PVA-CS-PANI-GSNO achieves spatiotemporal NO delivery under NIR control, which acts synergistically with MPTT to suppress inflammatory cytokine expression, promote ECM remodeling, and inhibit apoptosis of AF cells, ultimately facilitates the repair of the AF. By overcoming the limitations of systemic anti-inflammatory therapies imposed by the avascular nature of the AF, this strategy offers a promising avenue for the treatment of AF injuries associated with IVDD.

## Highlights

• A photothermal-responsive nanofibrous membrane for nitric oxide release was developed.
• Mild photothermal therapy synergizes with nitric oxide gas therapy to promote annulus fibrosus regeneration.
• The system suppresses inflammation, promotes extracellular matrix repair, and inhibits annulus fibrosus cell apoptosis.
• In vivo studies confirm improved intervertebral disc repair and restoration of biomechanical function.

## Graphical abstract

**Figure:**
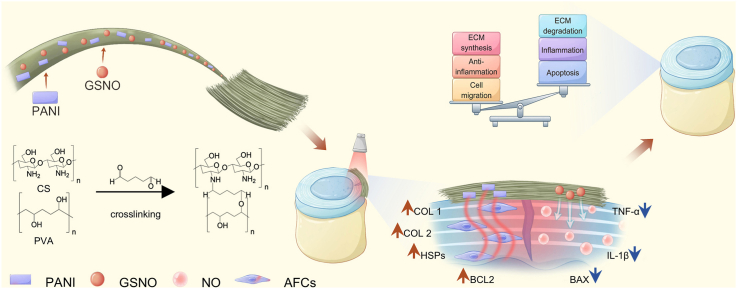

## 1 Introduction

Estimatedly, over 80 % of individuals encounter low back pain (LBP) during their lifetime. Among its primary etiologies, intervertebral disc degeneration (IVDD) is a leading cause [1]. The intervertebral disc (IVD) consists mainly of the nucleus pulposus (NP), annulus fibrosus (AF), and cartilage endplate. Dysfunction in any of these components can lead to IVDD [2]. Notably, AF plays a crucial role in preventing NP herniation and maintaining spinal stability [3]. However, during aging, the progression of IVD degeneration becomes inevitable [4]. In particular, inflammatory responses within the IVD are recognized as key pathophysiological changes driving IVDD, contributing not only to extracellular matrix (ECM) degradation but also to the downregulation of ECM synthesis [5,6]. As IVDD progresses further, fissures develop in the AF, allowing the unrestrained NP to protrude, ultimately resulting in discogenic LBP [7]. Therefore, controlling the impact of inflammation within the IVD and promoting the repair of the AF are of paramount importance.

Current conservative treatments for LBP primarily involve the oral administration of anti-inflammatory drugs, aiming to alleviate symptoms through their anti-inflammatory and analgesic properties [8]. However, due to the physiological structure of the IVD, which lacks a direct blood supply, systemic drug administration fails to achieve effective local drug concentration and therapeutic efficacy within the IVD [9]. Nitric oxide (NO), an endogenous anti-inflammatory mediator, has been widely utilized in gaseous anti-inflammatory therapy [10] and has been shown to promote fibroblast proliferation and migration, thereby facilitating fibroblast-mediated tissue healing [11]. As a key regulator of intracellular NO homeostasis, S-nitrosoglutathione (GSNO) is a naturally derived NO donor with excellent biocompatibility and has recently been investigated as an NO donor in NO gas therapy [12]. However, the rapid degradation of GSNO under free conditions limits its long-term applicability in vivo [13,14]. Fortunately, encapsulating GSNO within biomaterials can significantly reduce its degradation rate and regulate its controlled release [15].

Nanofiber scaffolds, which possess a structure similar to the natural ECM, provide a large specific surface area for cells, facilitating nutrient transport while enabling the efficient and low-adverse-effect encapsulation of therapeutic drugs [16,17]. Chitosan (CS) is widely used in biomedical engineering due to its antibacterial properties, biodegradability, and biocompatibility [18]. Electrospinning, recognized as the most suitable technique for nanofiber fabrication, offers advantages such as simplicity and cost-effectiveness [19]. With the assistance of polyvinyl alcohol (PVA), an electrospinning aid with excellent biocompatibility and biodegradability, CS can be electrospun into nanofibers to serve as a carrier for GSNO [20]. However, while nanofiber encapsulation can extend the storage stability of GSNO, it fails to achieve externally regulated controlled release.

Near-infrared (NIR)-mediated mild photothermal therapy (MPTT) promotes cell migration and ECM expression by inducing the expression of heat shock proteins (HSPs) and is regarded as an emerging approach for tissue repair [21]. This technique offers several advantages, including non-invasiveness, deep tissue penetration, high controllability, cost-effectiveness, and durability, making it a promising therapeutic strategy [22]. Among organic photosensitizers with excellent photophysical and photochemical properties, high photostability, and satisfactory biocompatibility, polyaniline (PANI) stands out as a particularly suitable candidate for NIR-mediated photothermal therapy due to its high photothermal conversion efficiency in the NIR spectrum [23]. Moreover, photothermal therapy can trigger the temperature-sensitive characteristics of GSNO, enabling on-demand NO release [24], which holds potential for synergistic integration with NO gas therapy to delay IVDD. However, to the best of our knowledge, no studies have yet explored the combined application of MPTT and photothermal therapy–mediated controlled NO gas therapy for IVDD treatment.

Here, we employed electrospinning technology to organically integrate PVA and CS while incorporating PANI, a biocompatible photothermal agent, and GSNO as an NO donor, thereby constructing the PVA-CS-PANI-GSNO (PCPG) composite nanofibrous membrane system. This system successfully achieved MPTT-induced controlled NO release, exerting anti-inflammatory effects while synergistically promoting cell migration and ECM expression, ultimately mitigating IVDD progression. We systematically evaluated the photothermal performance, controlled NO release, biocompatibility, and therapeutic efficacy of this composite nanofibrous membrane system for IVDD through in vitro, in vivo, and molecular biology experiments. The findings demonstrated that this composite nanofibrous membrane system exhibits excellent therapeutic potential for IVDD, offering a promising new treatment strategy.

## 2 Experimental section

### 2.1 Preparation of the electrospinning mixed solution

PANI rods were synthesized using our previously reported method [20]. 5 mg PANI was subsequently dispersed uniformly in 1 mL trifluoroethanol. Meanwhile, 5 mg of GSNO (Purity: 95 %, MACKLIN, Shanghai, China) was dissolved in 1 mL of ultrapure water. These two solutions were then combined with 8 mL of a 1 % (v/v) acetic acid in which 800 mg of PVA (PVA 1788, 90 % hydrolyzed, MACKLIN, China, with an average molecular weight of 6.72 × 10^4^ g/mol determined by viscometry) and 200 mg of CS (96.75 % deacetylated, the testing method for the degree of deacetylation is provided in the Supporting Information, MACKLIN, China, with an average molecular weight of 5.03 × 10^4^ g/mol determined by viscometry) had been dissolved, followed by stirring at 500 rpm for 10 h. The resulting mixture was the polymer solution prepared for electrospinning.

### 2.2 Preparation of the PCPG nanofibrous membrane

**Fig. 1:**
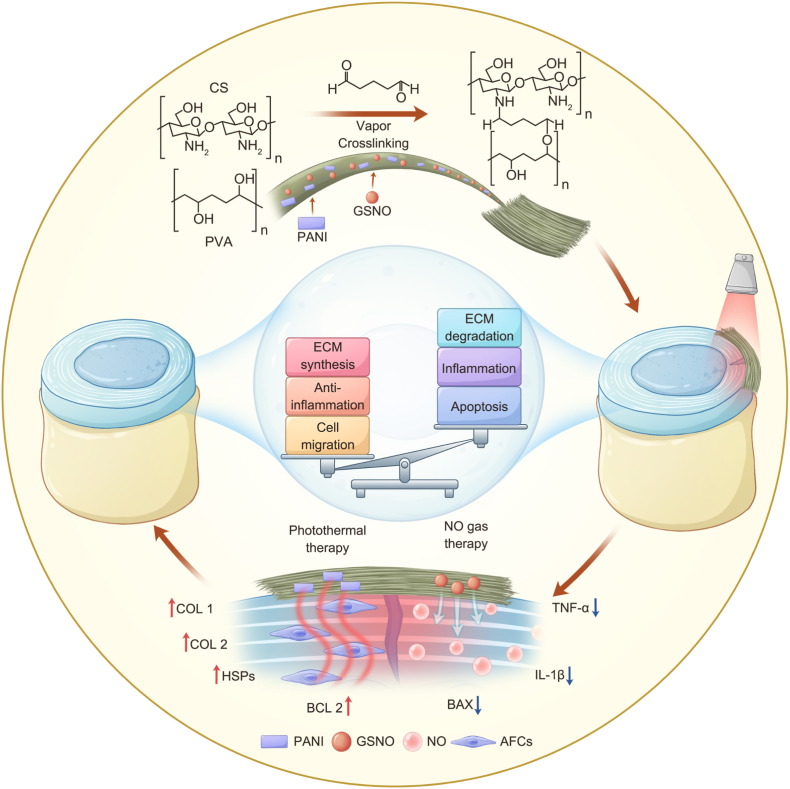
Illustration of the synthesis and therapeutic application of PCPG nanofibrous membranes.

Solution blends—PC (PVA/CS), PCG (with GSNO), PCP (with PANI), and PCPG (with both PANI and GSNO)—were electrospun and crosslinked under identical conditions. Specifically, 10 mL of the corresponding polymer solution was loaded into a syringe equipped with a 22 G stainless steel needle. The needle was linked to a 22 kV power source (NAYI, China), and the ground wire to a rotating drum (NAYI, China) collector (4000 rpm). The solution was delivered at 6 μL/min, with a 15 cm gap between the needle and the drum. The collected nanofibrous membranes were placed in a vacuum desiccator, where 2 mL glutaraldehyde (25 wt%) and 20 μL hydrochloric acid (37 wt%) were used as gaseous cross-linking agents. The pressure within the vacuum desiccator was set to 6 × 10^−2^ MPa. After 2 h of vapor-phase cross-linking, the nanofibrous membranes were transferred to a freeze-dryer (ZLGJ-10, KEWANGDA, China) and vacuum-dried for 8 h to ensure complete removal of residual glutaraldehyde and hydrochloric acid. The thickness of the PC-based nanofibrous membranes was determined using a micrometer (GREENER, China) to confirm sample uniformity. Fig. 1 illustrates the fabrication process of the nanofibrous membrane.

### 2.3 SEM characterization of nanofibrous membranes and PANI rods

Nanofibrous membrane microstructures were imaged using a scanning electron microscope (SEM) (Regulus8100, Hitachi, Japan). PC based nanofibrous membranes were mounted on aluminum stubs with conductive adhesive and sputter-coated with gold for 120 s at 10 mA. During SEM observation, elemental mapping of nitrogen was performed via energy-dispersive spectroscopy (EDS). Similarly, PANI rods were dispersed in trifluoroethanol on aluminum foil, dried, and analyzed using the same method. Finally, ImageJ (v1.54) was employed to analyze the nanofiber diameter and porosity of the nanofibrous membranes, as well as the ratio distributions of PANI rods.

### 2.4 FT-IR assay

A Fourier transform infrared (FT-IR) spectrometer (Nexus 670, NICOLET, America) was applied to analyze PVA, CS, and PC based nanofibrous membranes. Spectra were recorded from 4000 to 400 cm^−1^ using potassium chloride and ambient air as background references.

### 2.5 Wettability analysis

Wettability of the nanofibrous membranes was assessed via optical contact angle measurements (OCA20, Dataphysics, Germany). A 3 μL drop of deionized water was dispensed onto the surface of different samples, and static images were acquired via a high-resolution imaging system. Each sample was measured in triplicate, and the mean contact angle was reported.

### 2.6 Water absorption assay

After drying the nanofibrous membrane samples of the PC, PCG, PCP, PCPG, and PCPG + NIR (PCPG + N) groups in a vacuum drying oven for 12 h, the initial dry weight was recorded as M_0_. Subsequently, each group of samples was immersed in 10 mL of PBS, and the weight after 72 h was recorded as M_1_. Throughout the experiment, PCPG + N samples underwent on/off NIR irradiation (1 W/cm^2^, 5 min) at 0 and 48 h. The water absorption rate of the samples was calculated using the following formula:

(1) $$\begin{array}{c} water\;absorption\;ratio=\left[M_{1}{-M}_{0}\right]/\;M_{0} \end{array}$$

### 2.7 Degradation rate assessment of nanofibrous membranes

To evaluate the in vitro degradation rate of PC based nanofibrous membranes, samples (initial mass: W_0_) were immersed in 10 mL of PBS, and maintained at 37 °C for 8 weeks. Samples were retrieved weekly, and their water content was removed using a freeze-dryer before being weighed, with the mass recorded as Wx. For the PCPG + N group, samples underwent NIR on/off irradiation cycles every two days. The degradation rate was determined according to the following formula:

(2) $$\begin{array}{c} degradation\;rate=\left[\left(W_{0}-W_{x}\right)/W_{0}\right]\times 100\% \end{array}$$

Additionally, samples from the PCPG + N group were subjected to SEM observation following the completion of the in vitro degradation experiment to characterize their microstructural alterations.

### 2.8 Photothermal performance of nanofibrous membranes

Using an 808 nm laser (808, LEIRUI, China), samples from the PBS, PCG, and PCPG groups were irradiated at a power density of 1 W/cm^2^ for 120 s each. Considering the controllability of temperature elevation, as shown in Fig. S1h, NIR irradiation at 1 W/cm^2^ provided an optimal balance between efficient heating and temperature control under different power densities. To determine the power density of the near-infrared laser on the nanofibrous membranes, the laser output power was set to 1 W, and the distance between the fiber collimator and the target was adjusted to produce a spot with a diameter of 1.13 cm, corresponding to a spot area of 1 cm^2^. Thus, the power density was maintained at 1 W/cm^2^. An infrared thermal camera (UTi120S, UNI-T, China) was used to monitor temperature changes and evaluate the photothermal performance of the nanofibrous membranes. To evaluate the photothermal stability of the samples, the PCPG group was irradiated with the same near-infrared laser specifications for 25 s per cycle, with each subsequent cycle initiated only after the temperature had returned to 25 °C. In subsequent experiments, this NIR photothermal on/off cycling irradiation protocol was consistently applied to mitigate potential hazards of sustained high temperatures on cells and animals. Each treatment session lasted for 5 min.

### 2.9 NIR-responsive NO release from PCPG

PCPG nanofibrous membranes and an equivalent amount of free GSNO were separately incubated in 2 mL of PBS at 37 °C for 14 days, and cumulative NO release from each group was quantified at predefined time points using the Griess reagent (G4410-10G; Sigma-Aldrich). In brief, 50 μL of PBS incubated with the nanofibrous membrane was collected at specified time points and combined with an equal volume of Griess reagent in a 96-well microplate. After a 10-min dark incubation at 37 °C, absorbance at 540 nm was recorded with a microplate reader (Multiskan FC, ThermoFisher, China). NO release was quantified by comparison with a sodium nitrite standard curve obtained under identical conditions. To verify the NIR-induced NO release under controlled conditions, 10 mg of PCPG nanofibrous membrane samples were immersed in 2 mL of PBS and exposed to a 5-min on/off NIR light cycle every 55 min. The NO concentration in the PBS at different time points was measured using the Griess reagent.

### 2.10 Cell culture of annulus fibrosus cells

We acquired human annulus fibrosus cells (AFCs) from Procell (CP-H174, Procell, China) and cultured with the AFCs complete culture medium (CM-H174, Procell, China). Nanofibrous membranes were placed in transwell inserts (3 μm pore size, CORNING) and co-incubated with cells cultured in the lower chamber at 37 °C and 5 % CO_2_. The cells used in this study were at passage three. To establish the in vitro IDD model, AFCs were treated with 100 μg mL^−1^ Lipopolysaccharide (LPS, Solarbio, L8880) for 4 h. In the cell experiments, the experimental groups including Ctrl, LPS, PC, PCG, PCP, and PCPG were divided into NIR exposure groups and non-NIR exposed groups as required. NIR groups were exposed to cyclic 808 nm NIR irradiation at 1 W/cm^2^. Each irradiation lasted for 30 s, and after cooling to 37 °C, the next irradiation session was performed. The total duration of each NIR on/off cyclic irradiation treatment was 5 min. In the corresponding cell experiments, starting from day 1, NIR on/off cyclic irradiation was applied once every two days.

### 2.11 Cell proliferation and viability assays

The CCK-8 assay was used to evaluate the cellular proliferation activity of AFCs. Briefly, 1 × 10^4^ cells were co-cultured with PC based nanofibrous membranes for 1 or 3 days. Supernatant absorbance at 450 nm was measured using a microplate reader (Multiskan FC, ThermoFisher, China), with culture medium, Ctrl group, and treatment groups denoted as OD_b_, OD_c_, and OD_t_, respectively. Cell viability was determined using the formula below:

(3) $$\begin{array}{c} cell\;viability=\left({OD}_{t}-{OD}_{b}\right)\;/\;\left(OD_{c}{-OD}_{b}\right) \end{array}$$

The loading ratios of PANI and GSNO were determined by ensuring cell viability above 80 %, as validated by the CCK-8 assay.

The live/dead cell fluorescence assay was used to assess cytotoxicity and cell viability of AFCs under different treatments [25]. Following treatment, co-cultured AFCs were stained with a live/dead fluorescence kit (Bestbio, China), incubated in the dark for 20 min, and subsequently visualized under a fluorescence microscope (ECLIPSE Ti2-E, Nikon, Japan). For each group, three biological replicates were prepared, and fluorescence images were acquired from three randomly selected fields of view per sample. Quantitative analysis was performed using ImageJ software (v1.54), where green fluorescence represented live cells, counted as N _live_, and red fluorescence represented dead cells, counted as N _dead_. The cell viability was then calculated using the following formula:

(4) $$\begin{array}{c} {cell\;viability=N\;}_{live}\;/\;\left(N_{live}+N_{dead}\right) \end{array}$$

### 2.12 Scratch assay

The ability of nanofibrous membranes to promote AFCs cell migration was assessed using a scratch assay. 5 × 10^5^ AFCs were seeded in each well of a 6-well plate and cultured for 12 h until cell adhesion was achieved and the density reached 90 %. A scratch defect was then introduced using a 1 mL pipette tip. Cell migration was assessed on days 0 and 3 post-treatment using an optical microscope (Olympus, Japan).

### 2.13 Real time quantitative polymerase chain reaction (RT-qPCR)

AFCs were lysed, and total RNA was extracted using TRIzol reagent (ThermoFisher, Cat. No: 15596026CN). RNA purity and concentration were assessed with a NanoDrop One spectrophotometer (ThermoFisher, America). To target mRNA expression, total RNA was first digested with RNase R to remove linear transcripts, followed by reverse transcription into cDNA using the PrimeScript RT reagent kit (TaKaRa, Cat. No: RR037A). RT-qPCR was performed using the ABI 7500 real-time PCR system (ThermoFisher, America) with TB Green Premix Ex Taq II (TaKaRa, Cat. No: RR820A). Primer sequences are listed in Table S1, and GAPDH served as the internal reference gene. Relative mRNA levels were determined using the 2^−^ΔΔCT method. Each group included three biological replicates.

### 2.14 Western blot analysis

AFCs were lysed in RIPA buffer supplemented with 1 % PMSF, 1 % protease inhibitor, and 1 % phosphatase inhibitor (Beyotime, Shanghai, China) to extract total protein. Protein levels were quantified with a BCA assay kit (Beyotime, Shanghai, China). Proteins were denatured by heating at 100 °C for 15 min. Following SDS–PAGE separation (Epizyme), the proteins were transferred to PVDF membranes (MilliporeSigma, Darmstadt, Germany). Membranes were incubated in skim milk at room temperature for 1 h to block nonspecific binding, followed by incubation with the following diluted primary antibodies: HSP70(1:1000, ab181606, Abcam), HSP47(1:1000, ab109117, Abcam), COL1 (1:1000, ab270993, Abcam), COL2 (1:1000, Abclonal, RP02991), BCL2 (1:1000, ab117115, Abcam), BAX (1:1000, ab32503, Abcam), IL-1β(1:1000, Proteintech, 26048-1-AP), TNF-α(1:1000, Proteintech, 17590-1-AP), and GAPDH (1:50000, Proteintech, 60004-1-Ig). Following incubation, membranes were washed three times with TBST (Servicebio, Wuhan, China), followed by incubation with corresponding secondary antibodies at room temperature for 2 h. Detection of chemiluminescence was accomplished using an advanced chemiluminescent reagent (UElandy, Product Code: S6009M), and the resultant signals were captured using the Bio-Rad ChemiDoc Touch imaging system (America). The relative expression levels of proteins were quantified by analyzing band intensity with ImageJ.

### 2.15 Immunofluorescence assay

Each well of a 24-well plate was inoculated with 2 × 10^4^ AFCs. Following a 3-day incubation, cells were rinsed with PBS (Servicebio, Wuhan, China), fixed in 4 % paraformaldehyde for 15 min, and treated with 0.1 % Triton X-100 for permeabilization. Samples were incubated with 3 % BSA (Solarbio, Beijing, China) at room temperature for 1 h to block nonspecific binding, followed by overnight incubation at 4 °C with the primary antibodies listed below: COL1 (1:1000, ab270993, Abcam), COL2 (1:800, Proteintech, 28459-1-AP), HSP70(1:50, ab181606, Abcam), TNF-α(1:500, ab307164, Abcam). Samples were then exposed to Goat Anti-Rabbit IgG H&L secondary antibody conjugated to Alexa Fluor® 488 (1:200; ab150077, Abcam) for 1 h at room temperature. Nuclear staining was performed using DAPI (Beyotime). Fluorescence imaging was performed using a fluorescence microscope (Nikon Eclipse C1, Japan). Quantitative analysis of images was performed with ImageJ software.

### 2.16 Flow cytometry assay

Apoptosis in AFCs was assessed using the Annexin V-FITC/PI staining method (MultiSciences) following the manufacturer's protocol. Cells were washed twice with PBS and resuspended in 100 μL binding buffer (1 × 10^6^ cells/mL). After adding 5 μL Annexin V-FITC and 10 μL propidium iodide (PI), samples were incubated for 5 min at room temperature. Stained cells were analyzed by flow cytometry (Beckman Coulter, Fullerton, CA). The apoptotic index was defined as the percentage of PI- and Annexin V-positive cells within the total population.

### 2.17 Rat tail model of AF injury

All animal experiments were approved by the Ethics Committee of Nanchang University (Approval No. NCULAE-20221031076) and performed in strict accordance with the National Research Council's Guide for the Care and Use of Laboratory Animals. Male Sprague-Dawley rats aged 10–12 weeks were divided into six groups (Each group consisted of six rats): sham, defect, PCP, PCP + N, PCPG, PCPG + N. AF defects were induced in rat tail IVDs via needle puncture. Anesthesia was induced by intraperitoneal injection of 0.5 % pentobarbital sodium. The Co7/Co8 IVDs in the rat tail were selected for surgical intervention. After disinfection, a midline incision was made over the targeted disc region, and an 18G needle was used to create a full-thickness AF defect. Then, the various composite nanofibrous membranes were implanted into the defect site. The membrane can be directly implanted into the defect site using forceps, exhibiting good conformity without the need for sutures or adhesives, as shown in Fig. S7. Muscle and tendon tissues surrounding the defect were sutured, and the incision was closed. To prevent postoperative infections, the surgical sites of all rats were subjected to dressing changes and povidone-iodine disinfection every two days until complete wound healing. Rats were euthanized at 4 and 8 weeks after surgery, and IVD samples were harvested. During in vivo experiments, the NIR-treated group received intermittent 808 nm laser irradiation (1 W/cm^2^) in on/off cycles. Each irradiation lasted for 30 s, and the next irradiation session was performed after cooling to 37 °C. The total duration of each NIR on/off cyclic irradiation treatment was 5 min. Starting from day 1, NIR on/off cyclic irradiation was applied once every two days.

### 2.18 X-ray and MRI assay

Radiographic imaging of rat tails was performed at 4 and 8 weeks after surgery using a digital X-ray system (RV-20A, DAWEI, China). Disc height index (DHI) was calculated based on established protocol [26]. Rat caudal spines were imaged coronally using T2-weighted magnetic resonance imaging (MRI) on a 4.7 T system (BioSpec 47/30, Bruker, America). Grayscale intensity of the nucleus pulposus region was quantified via ImageJ to reflect its relative water content. IVD measurements were independently conducted by two experienced clinicians.

### 2.19 Histological analysis via tissue staining

At predefined time points, rat tail segments and major organs (heart, liver, spleen, lungs, and kidneys) were harvested, fixed in 4 % paraformaldehyde, and decalcified using an EDTA-based protocol for 3 weeks. Following dehydration, samples were paraffin-embedded and sliced at 4 μm thickness using a rotary microtome (Leica, Heidelberg, Germany). Tissue sections were subjected to hematoxylin and eosin (H&E) and safranin O/fast green (SO/FG) staining using established protocols. Histological degeneration of IVDs was evaluated based on a previously described grading system [27]. For immunohistochemical (IHC) staining, sections were incubated with the following primary antibodies: COL1 (1:1000, ab270993, Abcam), COL2 (1:800, Proteintech, 28459-1-AP), IL-1β (1:100, Proteintech, 26048-1-AP), TNF-α (1:1000, ab307164, Abcam). After primary antibody incubation, sections were exposed to matched secondary antibodies at 37 °C for 1 h. IHC signals were developed with a DAB substrate kit (Servicebio, G1212) and counterstained with hematoxylin (Servicebio, G1040). Quantitative analysis of images was performed with ImageJ.

### 2.20 RNA sequencing and downstream data analysis

Total RNA was isolated with TRIzol reagent (Invitrogen) according to the manufacturer's protocol and submitted to HaploX Genomics (Shenzhen, China) for high-throughput sequencing using the Illumina NovaSeq 6000 platform. Gene Set Enrichment Analysis (GSEA) was conducted to explore pathway enrichment differences across groups. Venn diagrams and heatmaps of normalized gene expression were visualized using TBtools.

### 2.21 Biomechanical tests of IVDs

Biomechanical properties of spinal segments were evaluated by axial loading before and after IVD injury, both in the presence and absence of repair [28]. Axial biomechanical testing was conducted using a universal material testing system (CMT6103, MTS, America). Cyclic loading was applied to the samples at 0.5 Hz for 20 cycles under force control at 25 °C. Compressive stiffness, tensile stiffness, range of motion (ROM), and axial neutral zone (NZ) length were evaluated based on the final complete loading cycle. Stiffness values were calculated based on the slope of the top 20 % segment within the linear region of the force–displacement curve. ROM was defined as the total axial displacement between peak compression and peak tension. NZ parameters were calculated using the Stiffness Threshold method [29] where NZ stiffness and length were defined as the slope and displacement of the neutral zone.

### 2.22 Statistical analysis

All statistical analyses were performed using Prism 8.0 software (GraphPad, America). Group comparisons were undertaken via one-way analysis of variance, with Bonferroni correction applied for multiple comparisons. Data are presented as mean ± standard deviation. Statistical significance was defined as ∗P < 0.05, ∗∗P < 0.01, ∗∗∗P < 0.001, ∗∗∗∗P < 0.0001.

## 3 Results

### 3.1 Construction and morphological characterization of nanofibrous membranes

#### 3.1.1 Morphological characterization of PANI rods and nanofibrous membranes by SEM

**Fig. 2:**
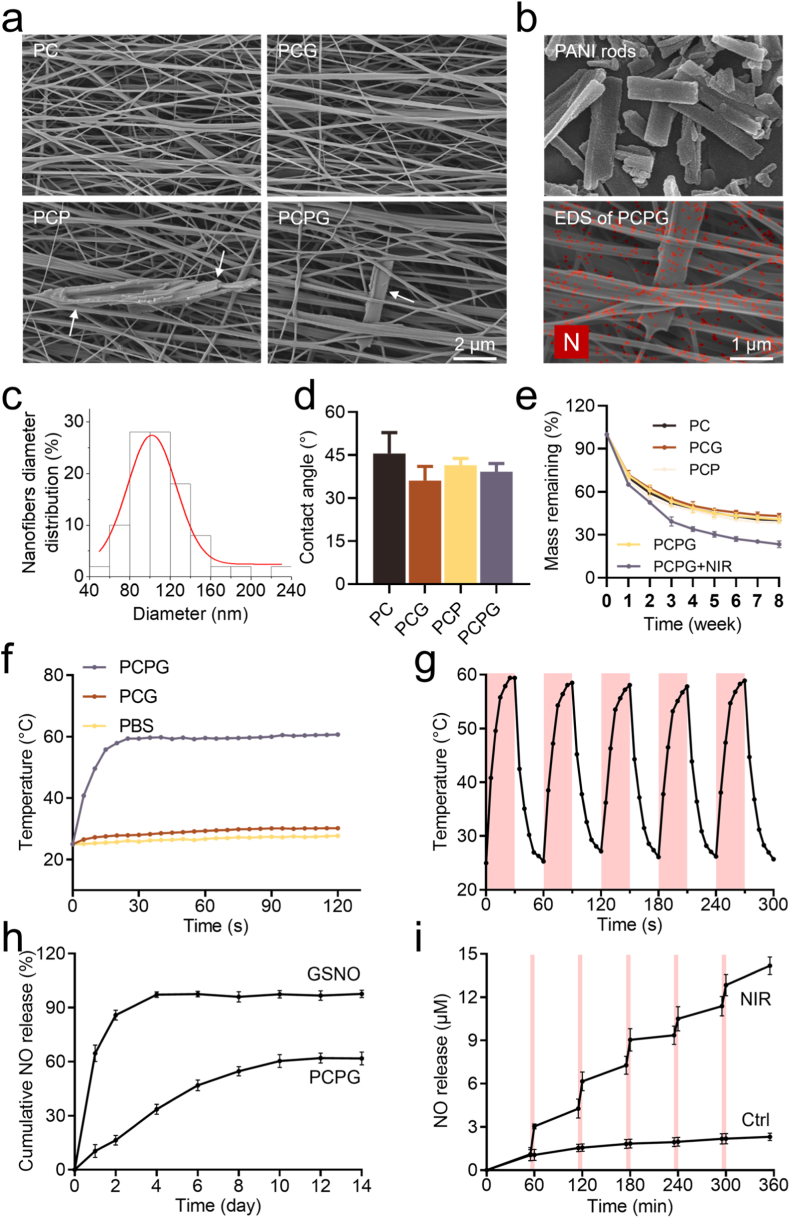
Characterization of PC based nanofibrous membranes. (a) SEM images of PC, PCG, PCP, and PCPG nanofibrous membranes. The white arrows indicate the PANI rods embedded within the nanofibrous membranes. (b) SEM image of PANI rods and elemental mapping of nitrogen on the PCPG nanofibrous membrane. (c) Diameter distribution of nanofibers in the PCPG nanofibrous membrane. (d) Water contact angle analysis of PC, PCG, PCP, and PCPG nanofibrous membranes. (e) Degradation rates of nanofibrous membranes from PC, PCG, PCP, PCPG, and PCPG + N groups. (f) Temperature changes of PBS, PCG, and PCPG nanofibrous membranes under 808 nm NIR irradiation at 1 W/cm^2^. (g) Temperature fluctuations of PCPG under cyclic 808 nm NIR on/off irradiation at 1 W/cm^2^. (h) Cumulative NO release from PCPG nanofibrous membranes and free GSNO over 14 days. (i) NO release responsiveness of the PCPG nanofibrous membrane under cyclic 808 nm NIR on/off irradiation at 1 W/cm^2^.

The PANI rods exhibited a relatively uniform rod-like structure under SEM observation (Fig. 2b), with a major axis length of 1.76 ± 0.68 μm and a minor axis length of 0.50 ± 0.19 μm (Fig. S1a and b). Additionally, the nanofibrous membranes electrospun from an 80 %:20 % (w/w) PVA:CS ratio displayed smooth nanofiber morphology (Fig. 2a). Under the influence of a high-speed drum collector operating at 3000 rpm, the nanofibers were aligned in a roughly parallel distribution. The diameters of the nanofibers in the PC, PCG, PCP, and PCPG groups were 93.82 ± 32.69 nm, 96.84 ± 33.86 nm, 126.31 ± 48.02 nm, and 112.33 ± 31.70 nm, respectively (Fig. 2c and Fig. S1c–e). PANI was uniformly embedded within the nanofibrous membranes, and the remaining regions of the membranes exhibited no significant structural alterations resulting from the incorporation of PANI and GSNO. As shown in Figs. S2 and S3, no statistically significant differences in thickness or porosity were observed among the PC-based nanofibrous membrane groups.

#### 3.1.2 FT-IR analysis

The Fourier-transform infrared (FT-IR) spectrum of the PCPG nanofibrous membrane exhibited multiple characteristic absorption peaks corresponding to the functional groups of its constituent components (Fig. S1g). The broad band around 3400 cm^−1^ is attributed to the O–H stretching vibrations of hydroxyl groups in PVA and CS, indicating their dominant presence in the system. The absorption peak at 1650 cm^−1^ corresponds to the C=O stretching vibration specific to CS. Bands near 1600 cm^−1^ and 1490 cm^−1^ are assigned to the aromatic C=C and C–N stretching vibrations of PANI, respectively. Notably, a characteristic absorption band around 1550 cm^−1^ corresponding to S–N stretching was observed, confirming the successful incorporation of GSNO into the system. The peak at 2920 cm^−1^ is a typical feature of C–H stretching vibrations from the alkyl chains in both PVA and PANI.

#### 3.1.3 Contact angle and water absorption assessment of nanofibrous membranes

As shown in Fig. 2d, the water contact angles of the PC based nanofibrous membranes were 45.56 ± 5.95°, 36.11 ± 4.07°, 41.52 ± 1.94°, and 39.21 ± 2.3°, respectively. No significant statistical differences were observed between the groups, indicating good hydrophilicity. Additionally, as illustrated in Fig. S1f, after 72 h of immersion, the mass of the nanofibrous membranes reached 290.00 ± 18.78 %, 288.67 ± 8.65 %, 299.00 ± 5.10 %, 305.33 ± 10.87 %, and 294.33 ± 5.19 % of the original mass. This suggests that the PC based nanofibrous membranes exhibit excellent water absorption capability.

#### 3.1.4 Degradation rate assessment of nanofibrous membranes

Fig. 2e shows that during the 8-week degradation experiment, the degradation rate of nanofibrous membranes followed a trend of initially fast degradation, which then slowed over time. By the second week, the remaining mass reached 51–64 % of the original mass. Subsequently, the degradation rate gradually slowed down, stabilizing in the 7th and 8th weeks with a remaining mass of 38–44 % of the original mass. Notably, the nanofibrous membrane samples from the PCPG group exhibited a faster degradation rate after undergoing NIR irradiation switch cycles, ultimately stabilizing at 23.47 ± 1.86 % of the initial mass by the 8th week. Moreover, Fig. S1j shows that the PCPG + N group maintained the fundamental architecture of the nanofibrous membrane after 8 weeks, indicating the durability of the PCPG nanofibrous membranes.

#### 3.1.5 Photothermal activity of nanofibrous membranes

Fig. S1h presents the temperature variation profiles of PCPG nanofibrous membranes under NIR irradiation at power densities of 0.5, 1, and 2 W/cm^2^. PCPG can achieve temperatures of 41.0, 59.4, and 109.8 °C within 30 s, respectively. Considering the effectiveness, controllability, and safety of the temperature rise, a power density of 1 W/cm^2^ with an 808 nm wavelength NIR was selected for subsequent experiments.

We evaluated and compared the photothermal performance of PCPG. Upon NIR exposure at 1 W/cm^2^, the temperature rapidly rose to approximately 60 °C within 30 s and reached a steady state by 120 s (Fig. 2f). In contrast, the PBS and PCG groups gradually reached temperatures of 27 °C and 30 °C, respectively, within the same period. The photothermal stability and controllability of PCPG nanofibrous membranes are evaluated in Fig. 2g. Under repeated NIR on/off irradiation every 30 s, with each irradiation lasting 30 s, the PCPG nanofibrous membranes can rapidly heat up to a level of 56.8–58.9 °C during irradiation. Upon cessation of irradiation, the temperature quickly drops back to the ambient temperature of 25 °C.

#### 3.1.6 NO release from PCPG in response to NIR irradiation

At 37 °C, compared with the free GSNO group, GSNO in PCPG maintained NO release extended to 12–14 days (Fig. 2h), providing a prolonged NO reservoir for early-stage photothermal and NO synergistic therapy. Fig. 2i also demonstrates the controllability of NO release from PCPG under NIR on/off irradiation. In comparison to the PCPG samples without NIR on/off irradiation, the PCPG samples subjected to NIR on/off irradiation can release NO in a controlled manner, induced by the photothermal effect of NIR.

### 3.2 Cell viability and biocompatibility assay

Cell proliferation assays Fig. S4a–c revealed that the PC based nanofibrous membranes did not induce cytotoxic effects. The cell viability/death staining results of AFCs Fig. S4d, e also demonstrated that the survival rate of AFCs co-cultured with the PC based nanofibrous membranes remained above 95 %. Over the 3-day culture period, AFC proliferation increased by 2.22–2.27-fold on average, suggesting that PC nanofibrous membranes were non-cytotoxic and supported cell viability.

### 3.3 AFCs exhibit intrinsic thermosensing ability

**Fig. 3:**
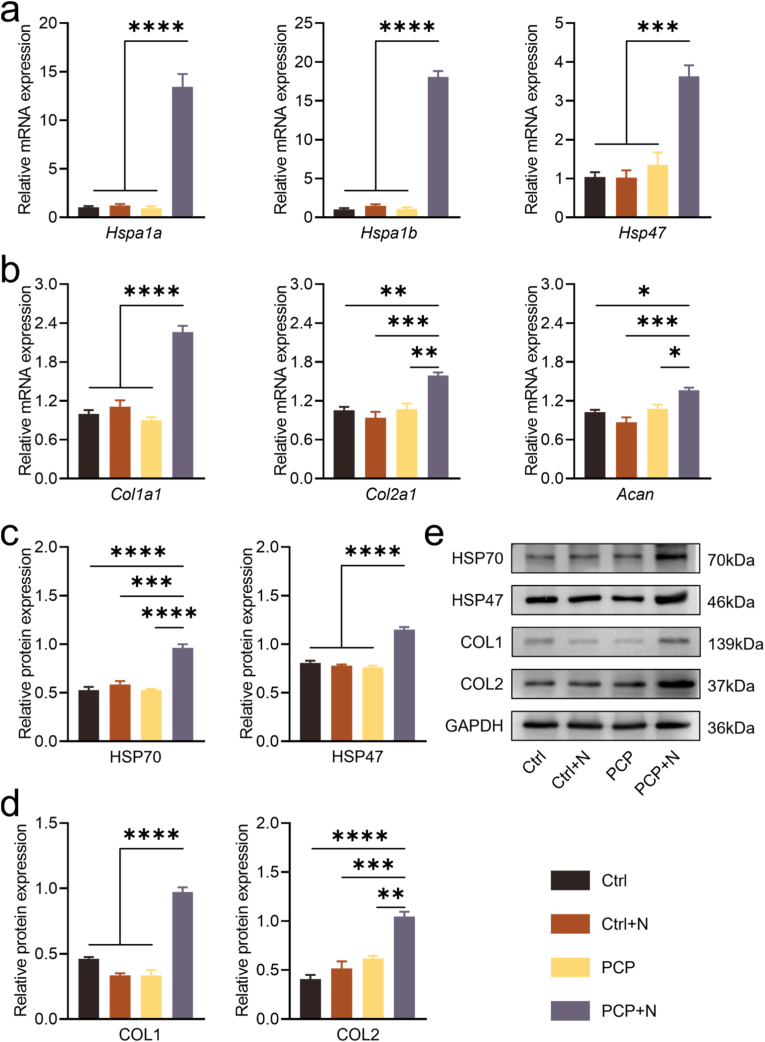
Regulation of gene and protein expression in AFCs (Ctrl, Ctrl + N, PCP, PCP + N) under photothermal therapy intervention. (a) HSP-related gene expression. (b) Expression of anabolic genes. (c, d) Densitometric quantification of Western blot results. (e) Protein levels of HSP- and anabolism-associated markers. (n = 3, ∗P < 0.05, ∗∗P < 0.01, ∗∗∗P < 0.001, and ∗∗∗∗P < 0.0001).

To assess the impact of mild hyperthermia, AFCs cultured with nanofibrous membranes were intermittently exposed to NIR irradiation. RT-qPCR analysis revealed a significant upregulation of HSP gene expression in AFCs upon photothermal stimulation (Fig. 3a). In the PCP + N group, matrix synthesis–associated genes including Col1a1, Col2a1, and Acan were significantly upregulated relative to the control (Fig. 3b). Notably, NIR exposure alone, in the absence of a photothermal agent, induced negligible changes in gene expression. Protein-level validation via Western blotting confirmed similar trends in HSP and anabolic marker expression across groups (Fig. 3c–e).

### 3.4 In vitro evaluation of combined photothermal and NO gas therapy

To evaluate treatment efficacy, an IVDD model was established by LPS stimulation in AFCs and subjected to photothermal and NO co-treatment. RT-qPCR results indicated that LPS stimulation upregulated inflammatory gene expression (Il1b, Tnf) in AFCs (Fig. S6c). Among all groups, the PCPG + N group showed the greatest downregulation of inflammation-associated genes. NIR stimulation enhanced HSP gene transcription in AFCs (Fig. S6a). In the PCPG + N group, genes associated with ECM synthesis (Col1a1, Col2a1, Acan) were markedly upregulated (Fig. S6b). Moreover, this group showed increased Bcl2 expression accompanied by reduced Bax transcription (Fig. S6d).

**Fig. 4:**
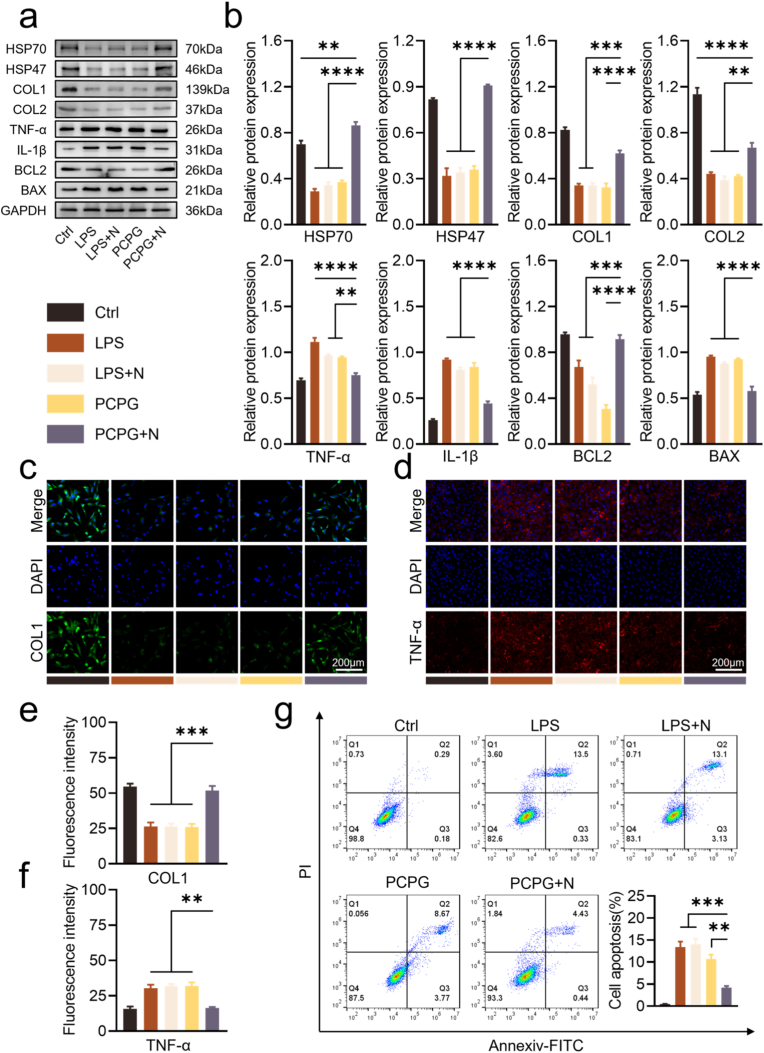
Protein expression of AFCs in Ctrl, LPS, LPS + N, PCPG, PCPG + N groups. (a) Protein levels related to HSPs, anabolism, inflammation, and apoptosis in AFCs. (b) Densitometric quantification of Western blot bands. (c) Immunofluorescence (IF) staining of COL1. (d) IF staining of TNF-α. (e, f) Quantification of IF signals. (g) Apoptosis analysis of AFCs by flow cytometry. (n = 3, ∗∗P < 0.01, ∗∗∗P < 0.001, and ∗∗∗∗P < 0.0001).

Next, protein-level changes in AFCs following synergistic treatment were evaluated to determine therapeutic efficacy (Fig. 4a and b). Among all groups, NIR-triggered PCPG + N treatment resulted in the most elevated levels of HSP-related proteins. Also, COL1 and COL2 protein expression was markedly elevated in the PCPG + N group compared to other treatment groups. Additionally, compared to the other intervention groups, the PCPG + N group exhibited the lowest expression of inflammation-related proteins, such as IL-1β and TNF-α. Beyond the suppression of inflammatory proteins, the PCPG + N group exhibited upregulation of anti-apoptotic markers and downregulation of pro-apoptotic proteins. Similarly, immunofluorescence staining confirmed trends observed in both Western blot and RT-qPCR results (Fig. 4c–f and Fig. S5a–d). Flow cytometric data revealed that apoptosis was most effectively inhibited in the PCPG + N group (Fig. 4g).

Finally, a scratch assay was conducted to verify the synergistic effect of photothermal and NO gas therapy on the promotion of AFCs cell migration. The PCPG + N group exhibited the most significant promotion of AFCs cell migration, with a migration rate of 68.80 ± 7.34 % after 3 days of co-incubation (Fig. S5e and f).

### 3.5 In vivo regeneration of IVD using nanofibrous membranes

**Fig. 5:**
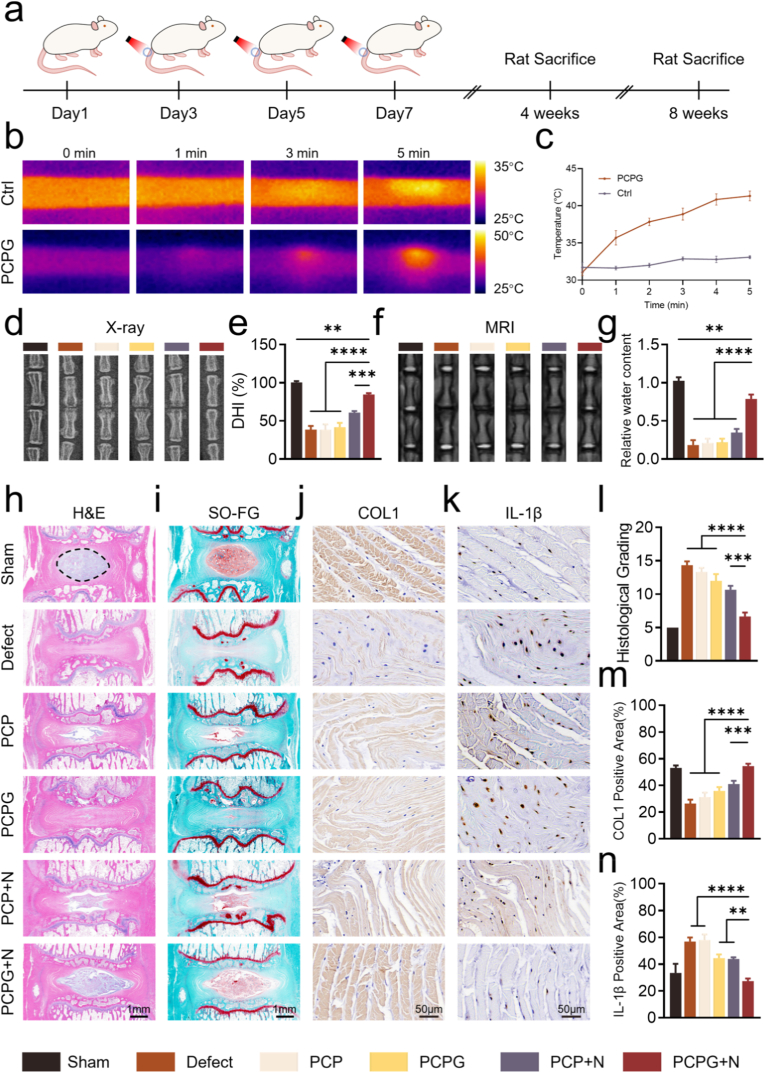
In vivo assessment of IVD regeneration using nanofibrous membranes. (a) Schematic of the experimental procedure. (b) Photothermal imaging of membranes in the rat caudal vertebra under 808 nm NIR irradiation. (c) Corresponding temperature profiles of the membranes. (d) X-ray images of rat caudal vertebrae at week 8. (e) DHI quantification. (f) MRI scans of rat caudal vertebrae at week 8. (g) Measurement of relative IVD water content. (h, i) Representative H&E and SO/FG staining of IVD sections from Sham, Defect, PCP, PCPG, PCP + N, and PCPG + N groups at week 8. Black dashed line denotes the AF–NP boundary. (j) Immunohistochemical (IHC) staining image of COL1 and IL-1β at week 8. (l) Histological grades. (m, n) Quantitative analysis of IHC staining. (n = 3, ∗∗P < 0.01, ∗∗∗P < 0.001, and ∗∗∗∗P < 0.0001).

To assess in vivo efficacy, nanofibrous membranes were applied to the IVD of rats following surgery. Based on in vitro screening, six groups were defined for in vivo experiments: Sham, Defect, PCP, PCPG, PCP + N, and PCPG + N. One hour post-surgery, animals assigned to the NIR treatment group were anesthetized with phenobarbital sodium. NIR irradiation was subsequently administered every other day throughout the experimental period (Fig. 5a).

In vivo photothermal responsiveness of nanofibrous membranes in the rat tail is a critical factor influencing their efficacy in IVDD repair. To assess the in vivo photothermal responsiveness of nanofibrous membranes in the rat tail, thermal images were captured with an NIR-sensitive infrared camera. Thermal imaging showed a distinct increase in temperature in the caudal vertebrae of rats implanted with PCPG membranes following cyclic NIR exposure, reaching 41.30 ± 0.65 °C within 5 min (Fig. 5b and c). By comparison, rats that did not receive PCPG membrane implantation showed minimal thermal response under NIR exposure.

IVD height and NP signal intensity were assessed by X-ray and MRI at 4 and 8 weeks following treatment. At week 4, except for the Sham group, the PCPG + N group showed the least reduction in IVD height and DHI (Fig. S8a and b). MRI signal intensity, indicative of IVD water content, was lowest in degenerated discs. At 4 weeks, excluding the Sham group, the PCPG + N group demonstrated the strongest NP signal, suggesting improved hydration and reduced degeneration (Fig. S8c and d). To assess the impact of NO and mild hyperthermia on IVD regeneration, histological and immunohistochemical analyses were performed. H&E staining revealed the morphological integrity and NP–AF boundary (Fig. S8e). In the Sham group, the NP remained intact with a clearly defined boundary, whereas the Defect group showed marked NP shrinkage and AF deformation at week 4. Although a slight decrease in NP area was noted in the PCPG + N group, the overall degree of degeneration remained relatively limited. SO/FG staining highlighted differences in collagen and proteoglycan distribution (Fig. S8f), with the PCPG + N group retaining the highest matrix content among the treated groups, second only to the Sham. The aforementioned observations were all in alignment with the histological grading (Fig. S8i). Immunohistochemistry confirmed these findings, showing decreased expression of pro-inflammatory markers IL-1β and TNF-α in the PCPG + N group (Figure S8h, k and Figure S8b, f), along with enhanced intracellular deposition of ECM proteins COL1 and COL2 (Figure S8g, j and Figure S9a, e).

At 8 weeks, radiographic analysis revealed varying degrees of vertebral damage in the Defect, PCP, PCPG, and PCP + N groups, characterized by irregular vertebral margins and further reduction in IVD height compared to the 4-week time point. In contrast, the PCPG + N group maintained relatively smooth vertebral borders and preserved disc height (Fig. 5d and e). MRI assessment showed pronounced loss of water content in the NP tissue of all groups except PCPG + N, where signal intensity remained high and hydration was largely retained (Fig. 5f and g). Histological evaluation via H&E and SO/FG staining confirmed better disc architecture in the PCPG + N group, including greater preservation of ECM, with enhanced collagen and proteoglycan content (Fig. 5h and i). The histological grading confirmed the earlier observations (Fig. 5l). Immunohistochemistry further supported these findings, showing more extensive COL1 and COL2 deposition in the PCPG + N group, in agreement with the histological results (Fig. 5j–m and Fig. S9c and g). Consistent with week 4 findings, IL-1β and TNF-α expression remained lowest in the PCPG + N group relative to all other groups (Fig. 5k–n and Figure S9d, h).

### 3.6 Transcriptomic analysis of differentially expressed mRNAs unveils the therapeutic mechanism for AFCs

**Fig. 6:**
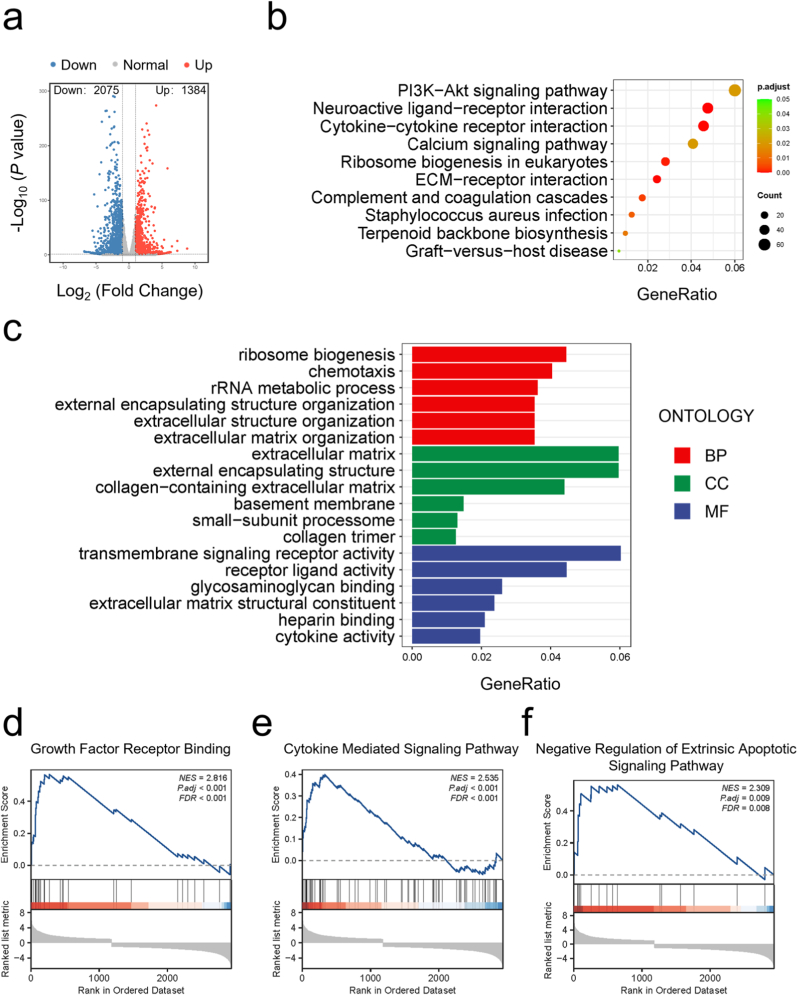
Molecular mechanisms of synergistic therapy. (a) Volcano plot displaying DEGs between the LPS and PCPG + N groups. (b) Perform GO enrichment analysis on differentially expressed. (c) KEGG enrichment analysis of the same DEG set. (d–f) GSEA enrichment analysis of differential genes.

To explore how AFCs respond to the co-application of photothermal therapy and NO, transcriptomic profiling was conducted via RNA sequencing following the respective interventions. The heatmap revealed distinct transcriptional profiles between the LPS and PCPG + N groups (Fig. S11a). A volcano plot was generated to illustrate DEGs between the LPS and PCPG + N groups (Fig. 6a). RNA-seq analysis identified 3459 DEGs in the PCPG + N group, including 1384 upregulated and 2075 downregulated transcripts. Gene Ontology (GO) and Kyoto Encyclopedia of Genes and Genomes (KEGG) enrichment analyses were conducted to clarify the biological processes involved (Fig. 6b and c). The GO analysis revealed strong associations between the differential genes and processes such as ECM structural constituent, collagen−containing ECM, cytokine activity. KEGG analysis identified multiple enriched pathways, with prominent involvement of PI3K-Akt signaling, cytokine–cytokine receptor interactions, and ECM–receptor interactions. Furthermore, GSEA highlighted several differentially enriched pathways, including growth factor receptor binding, cytokine mediated signaling pathway, negative regulation of extrinsic apoptotic signaling pathway, ribonucleoprotein complex biogenesis, growth factor activity and cytokine activity (Fig. 6d–f and Fig. S11b–d).

### 3.7 Mechanical evaluation of isolated rat caudal vertebrae

**Fig. 7:**
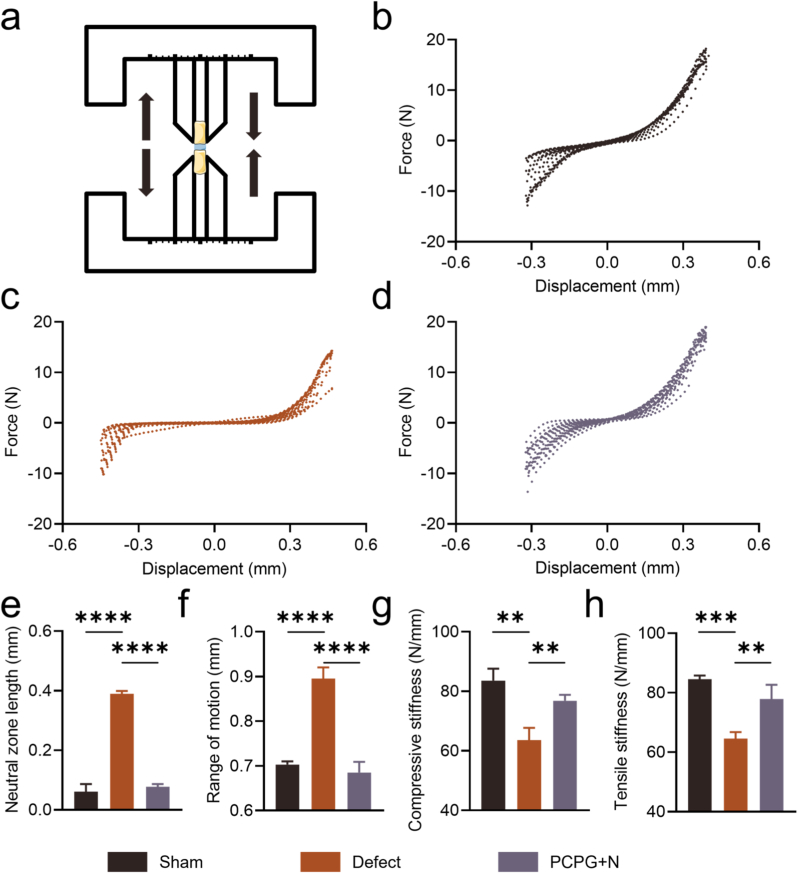
Biomechanical evaluation of IVDs. (a) Diagram illustrating cyclic loading applied to the IVD. (b–d) Representative force–displacement profiles from the Sham, Defect, and PCPG + N groups subjected to cyclic tensile–compressive loading. (e–h) Quantitative comparisons of torsional neutral zone length, range of motion, compressive modulus, and tensile modulus. (n = 3, ∗∗P < 0.01, ∗∗∗P < 0.001, and ∗∗∗∗P < 0.0001).

Biomechanical testing was performed to assess functional recovery of the IVD following treatment with the PCPG nanofibrous membrane (Fig. 7a). Axial cyclic tension–compression testing revealed that the Defect group exhibited significantly greater axial ROM and NZ length relative to the Sham group, indicative of pronounced disc degeneration. Notably, there was no significant increase in the ROM and NZ length in the PCPG + N group, which remained at a relatively low level. Meanwhile, both compressive and tensile stiffness were better maintained in this group, indicating a partial recovery of biomechanical performance (Fig. 7b–h).

## 4 Discussion

AF repair remains a formidable challenge due to its inherently low cellularity, limited metabolic activity, and exposure to a complex biomechanical environment. These factors, compounded by a localized pro-inflammatory microenvironment, severely hinder the tissue's intrinsic regenerative capacity. In recent years, tissue-engineering-based strategies for AF repair—such as biomaterial scaffolds and bioadhesive sealants—have garnered increasing attention, aiming to facilitate AF regeneration and ultimately delay IVDD [30,31]. In this study, we successfully fabricated a nanofibrous membrane via electrospinning technology, integrating PANI with GSNO. Driven by the combined action of photothermal therapy and NO gas therapy, the resulting membrane significantly enhanced ECM synthesis while suppressing the inflammatory microenvironment and apoptosis of AFCs, thereby promoting effective AF repair and regeneration.

As shown in Fig. 2a, owing to the excellent spinnability of PVA, CS and PVA were co-electrospun into uniform nanofibers. Under the influence of a high-speed rotating drum collector, the nanofibers exhibited an overall parallel alignment, closely mimicking the native fiber orientation observed in the outer annulus fibrosus. Benefiting from the versatility of the electrospinning process, PANI and GSNO were uniformly encapsulated within the nanofibrous membrane, providing a structural basis for the stable incorporation and preservation of GSNO. The resulting PCPG membrane displayed an intact, porous, and homogeneous architecture. Hydrophilicity and water absorption capacity are fundamental to the biocompatibility and drug delivery performance of biomaterials. As shown in Fig. 2d, the PC based nanofibrous membranes exhibited excellent hydrophilicity, which facilitates cell adhesion and proliferation. Swelling data (Fig. S1f) further revealed that the PC based membranes could absorb approximately three times their own weight in fluid, a property advantageous for sustained drug release and efficient therapeutic delivery. The biocompatibility of PCPG was validated using CCK-8 assays, live/dead cell staining, and H&E staining of major organs (Figs. S4 and S10). Results indicated that the PCPG nanofibrous membrane exhibited no apparent cytotoxicity or systemic toxicity.

MPTT minimizes thermal injury, enhances local perfusion and nutrient delivery, promotes ion channel activation and mobility, and triggers HSPs expression [32]. In photothermal therapy, HSPs mediate tissue regeneration, with HSP70 induction protecting against ischemia-reperfusion injury, neurodegeneration, epilepsy-related brain damage, and muscle injury [33]. HSP47 functions as a collagen-targeted chaperone required for the proper folding and maturation of type I and II collagen molecules [34]. Collagen maintains IVD stability under stress, with HSP47 aiding ECM deposition. Our study showed photothermal therapy upregulates HSPs and ECM synthesis in AFCs (Fig. 3), suggesting photothermal therapy-induced HSPs may offer therapeutic potential for IVDD.

Inflammation is one of the primary factors hindering the self-repair of AF injuries. [35]. As a dense, avascular connective tissue [36], the AF exhibits inherently low permeability, which has prompted increasing interest in gas therapy as a promising intervention. Owing to its exceptional diffusibility, NO has emerged as a key regulator in promoting tissue regeneration [37]. To overcome the instability of GSNO, it was encapsulated within nanofibrous membranes. The PCPG nanofibrous membranes exhibited excellent GSNO retention (Fig. 2h) and enabled highly controllable, thermally responsive release of NO (Fig. 2i). Experimental results (Fig. 4) demonstrated that under the synergistic effect of MPTT-NO gas therapy, there was an upregulation of heat shock proteins and collagen synthesis markers in AFCs, accompanied by a downregulation of inflammatory cytokines TNF-α and IL-1β. Collectively, these findings indicate that the dual MPTT-NO gas therapy effectively mitigates inflammation.

Apoptosis of AFCs is a critical factor in the progression of IVDD [38]. Following PCPG + N treatment, the expression of the anti-apoptotic gene Bcl2 was upregulated, while that of the pro-apoptotic gene Bax was downregulated. Flow cytometry analysis confirmed the anti-apoptotic effect of the therapy. Cellular migration plays a vital role in tissue repair [39]. Scratch wound assays revealed significantly enhanced migration of AFCs in the PCPG + N group, suggesting that photothermal-NO combination therapy facilitates cell motility. Collectively, this study highlights the therapeutic potential of photothermal-NO synergistic therapy for IVDD. By targeting multiple cellular processes—including inflammation modulation, ECM synthesis, apoptosis inhibition, and migration enhancement—this approach offers a multifaceted strategy to ameliorate the pathological progression of IVD degeneration.

Fig. 5 highlights the in vivo efficacy of the PCPG nanofibrous membrane in facilitating AF regeneration. Thermal imaging under NIR irradiation confirmed that the PCPG membrane achieved efficient photothermal conversion in the caudal disc region. Imaging analyses further revealed that the membrane preserved disc height to the greatest extent under photothermal stimulation and maintained high water content in the NP, thereby effectively suppressing degenerative progression. Histological analyses revealed that PCPG + N preserves disc architecture and matrix composition, with reduced NP loss and enhanced collagen and proteoglycan retention. Notably, inflammatory markers IL-1β and TNF-α were markedly suppressed, while increased COL1 and COL2 deposition indicates effective ECM reconstruction.

Transcriptomic sequencing revealed that PCPG + N treatment markedly reprogrammed gene expression compared to LPS-induced injury. GO enrichment analysis showed strong enrichment in functional categories such as ECM structural components, collagen-associated ECM, and cytokine activity. KEGG pathway analysis further identified significant enrichment in key signaling cascades, including the PI3K-Akt pathway, cytokine–cytokine receptor interactions, and ECM–receptor interactions. PI3K-Akt pathway activation promotes AFCs proliferation and protects against apoptosis, while also influencing ECM dynamics to support tissue homeostasis [40]. Simultaneously, GSEA reinforced these findings. For example, upregulation of growth factor receptor binding promotes matrix protein expression [41]. Taken together, this combinatorial therapy orchestrates a multi-level, multi-pathway regulatory response at the molecular level, promoting ECM regeneration, anti-inflammatory activity and inhibiting apoptosis—collectively contributing to the therapeutic repair of AF.

Slight structural disruptions within the IVD may markedly influence its biomechanical performance [42]. To evaluate the mechanical restoration following injury and treatment, we performed biomechanical evaluations for the sham, defect, and PCPG + N groups, respectively. As shown in Fig. 7, key parameters—including ROM, NZ length, compressive and tensile stiffness—were markedly improved in the PCPG + N group compared to the defect group, albeit still slightly inferior to the sham group. These findings indicate that after 8 weeks of treatment, PCPG + N group significantly restored the biomechanical function of the degenerated IVD.

Comprehensive in vivo and in vitro experiments demonstrate that the PCPG nanofibrous membrane, under the synergistic action of MPTT and NO therapy, effectively delays the progression of IVDD and exhibits strong therapeutic potential for functional AF regeneration. However, our study has certain limitations. The treatment of IVDD models or disease requires a prolonged duration. To achieve superior photothermal performance, we employed PANI as the photosensitizer, despite its limited biodegradability in vivo. Moreover, the long-term toxicity of PANI in vivo has not yet been thoroughly investigated, necessitating further assessment of its long-term biosafety.

## 5 Conclusion

In this study, we developed a multifunctional PCPG nanofibrous membrane system to address key challenges in AF repair, including the inflammatory microenvironment, impaired ECM synthesis, and restricted cellular migration. Upon NIR irradiation, the membrane enables MPTT by delivering localized heat deep into the damaged AF. Simultaneously, it activates the thermosensitive release of NO from GSNO, enabling controlled NO gas therapy. This dual-action system synergistically promotes cell migration while counteracting the inflammatory milieu. By fully leveraging the versatility of electrospinning technology, this therapeutic platform demonstrated robust efficacy and holds promise as a novel strategy for the treatment of AF injury. Moving forward, future research should place greater emphasis on optimizing photothermal parameters for deeper tissue applications to advance clinical translation.

## CRediT authorship contribution statement

**Guanfeng Huang:** Writing – review & editing, Writing – original draft, Validation, Software, Data curation. **Jiajun Xie:** Writing – original draft, Software, Data curation. **Jialan Chen:** Writing – original draft, Software, Data curation. **Jiangminghao Zhao:** Visualization, Investigation. **Pinkai Wang:** Visualization, Investigation. **Jian Zhang:** Visualization, Investigation. **Peichuan Xu:** Validation, Software. **Yang Li:** Validation, Software. **Xiaolong Chen:** Validation, Software. **Xinxin Miao:** Writing – review & editing, Funding acquisition. **Wei Xiong:** Supervision, Conceptualization. **Xigao Cheng:** Writing – review & editing, Supervision, Funding acquisition, Conceptualization.

## Declaration of competing interest

The authors declare that they have no known competing financial interests or personal relationships that could have appeared to influence the work reported in this paper.

## Data Availability

Data will be made available on request.
