# Supplementary material for: Photothermal-triggered NO-releasing nanofiber membrane mitigates intervertebral disc degeneration via inflammation inhibition and matrix stabilization
Source: Mater Today Bio. 2025 Sep 4;35:102287. doi: 10.1016/j.mtbio.2025.102287 (PMC12451378; doi:10.1016/j.mtbio.2025.102287)
Supplement: Multimedia component 1 [file mmc1.docx]

Supporting Information

*For*

**Photothermal-triggered NO-releasing nanofiber membrane mitigates intervertebral disc degeneration via inflammation inhibition and matrix stabilization**

Guanfeng Huang ^1,2#^, Jiajun Xie ^1,2#^, Jialan Chen^4#^, Jiangminghao Zhao ^1,2^, Pinkai Wang ^1,2^, Jian Zhang^1,2^, Peichuan Xu ^1,2^, Yang Li ^1,2^, Xiaolong Chen ^1,2^, Xinxin Miao^1,2,3^*, Wei Xiong ^1,2,3^*, Xigao Cheng ^1,2,3^*

^1^ Department of Orthopedics, The Second Affiliated Hospital, Jiangxi Medical College, Nanchang University, Nanchang, Jiangxi, 330006, China.

^2^ Jiangxi Provincial Key Laboratory of Spine and Spinal Cord Disease, Nanchang, Jiangxi, 330006, China.

^3^ Institute of Minimally Invasive Orthopedics, Nanchang University, Nanchang, Jiangxi, 330006, China.

^4^ Division of Orthopaedic Surgery, Department of Orthopaedics, Nanfang Hospital, Southern Medical University, Guangzhou, Guangdong, 510515, China.

^#^ These authors contributed equally to this work.

Correspondence: Xinxin Miao^1,2,3^*, Wei Xiong ^1,2,3^*, Xigao Cheng ^1,2,3^*

M.D. Xinxin Miao, E-mail: 453697735@qq.com

M.D. Wei Xiong, E-mail: weixiong@whu.edu.cn

Prof. Xigao Cheng, E-mail: xigaocheng@hotmail.com

***1. Methods***

***1.1 Determination of the degree of deacetylation (DD) of chitosan (CS) by ¹H NMR***

A 10 mg sample of chitosan was dissolved in 1.5 mL of DCl/D₂O solution (pH = 4) and stirred at room temperature for 24 hours. Subsequently, 0.7 g of the resulting solution was subjected to 14,000 scans using a nuclear magnetic resonance spectrometer (Bruker 400 MHz, Germany). The degree of deacetylation (DD) of CS was then calculated using the following formula:

$$\begin{aligned} \text{DA = ( }I_{CH3}\text{/3) / ( }I_{H2-H6}\text{/6) }\#\text{(S1)} \end{aligned}$$

$$\begin{aligned} \text{DD = (1-DA) × 100\%}\#\text{(S2)} \end{aligned}$$

Here, DA represents the degree of acetylation; I_CH3_ corresponds to the integral area of the methyl protons (–CH₃) of the acetyl group, located around 2.0 ppm; and I_H2-H6_ represents the total integral area of the H2 to H6 protons in the D-glucosamine backbone, typically observed in the region of 3.0–5.5 ppm.

***2. Results***

***2.1 ¹H NMR spectrum of chitosan and calculation results of DD***

The obtained ¹H NMR spectrum was analyzed and the integral areas were calculated using MestReNova 14.0 software. As shown in Figure S1i, ICH3 was 0.21 (corresponding to the region around 1.92 ppm), and IH2-H6 was 12.92 (corresponding to the 3–5 ppm region, excluding the integral area of D₂O). The resulting DD was calculated to be 96.75%.

***2.2 In vivo safety evaluation of PCPG nanofibrous membranes.***

As shown in Figure S10, no structural damage, cellular degeneration, or inflammatory infiltration was observed in major organs (heart, liver, spleen, lungs, and kidneys) after 8 weeks of treatment in the PCPG+N group, indicating the biosafety of PCPG nanofibrous membranes.

**
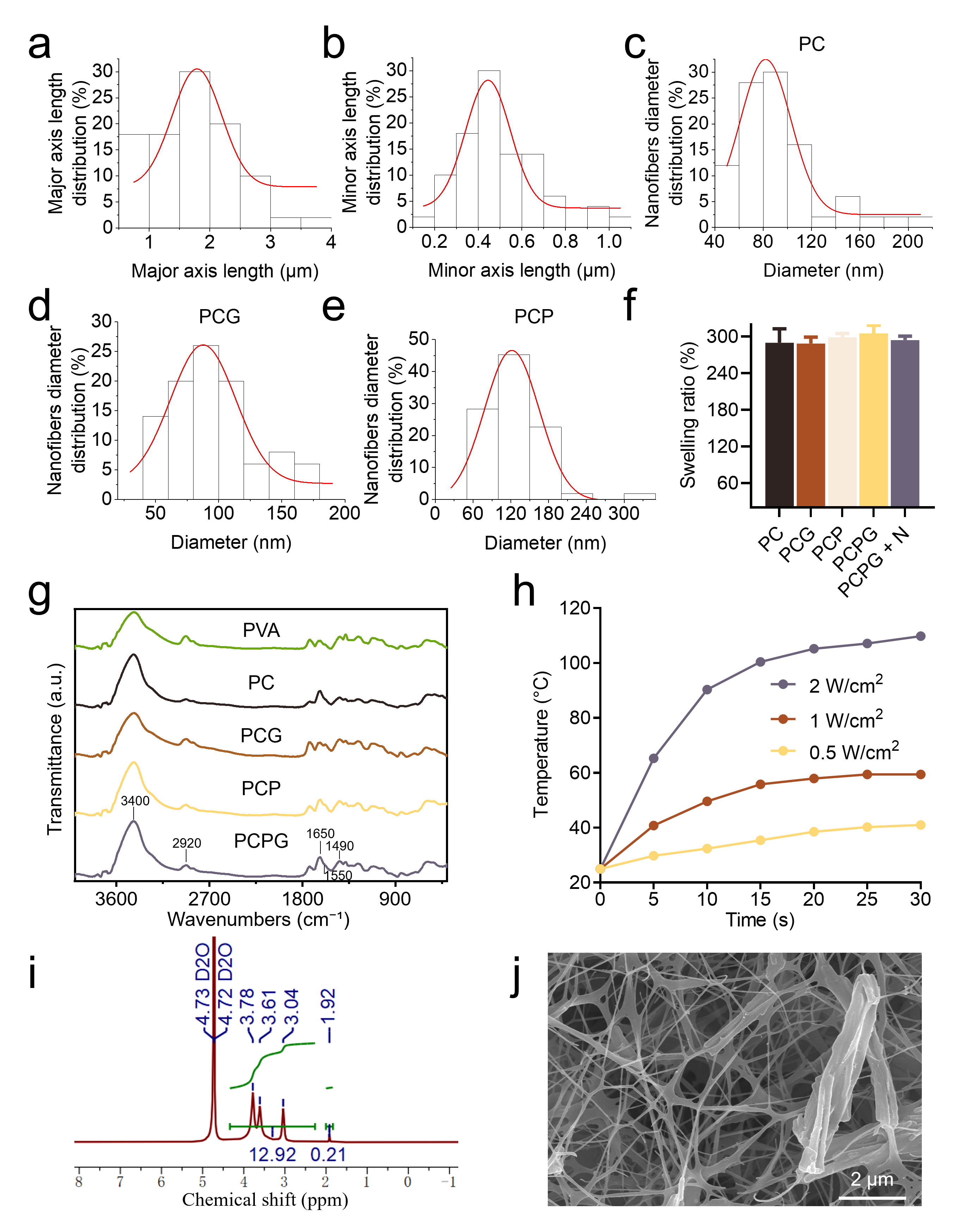
**

**Figure S1.** Characterization of PANI rods and PC based nanofibrous membranes. (a–b) Dimensional distribution of PANI rods along their major and minor axes. (c–e) Diameter distributions of nanofibers within PC, PCG, and PCP nanofibrous membranes. (f) Swelling ratios of nanofibrous membranes from the PC, PCG, PCP, PCPG, and PCPG+N groups. (g) FT-IR absorption spectra of nanofibrous membranes composed of PVA, PC, PCG, PCP, and PCPG. (h) Thermal response of PCPG membranes under 808 nm NIR exposure at power levels of 0.5, 1, and 2 W/cm². (i) ¹H NMR spectrum of CS. (j) Samples from the PCPG+N group retained the nanofibrous membrane structure even after 8 weeks of treatment.


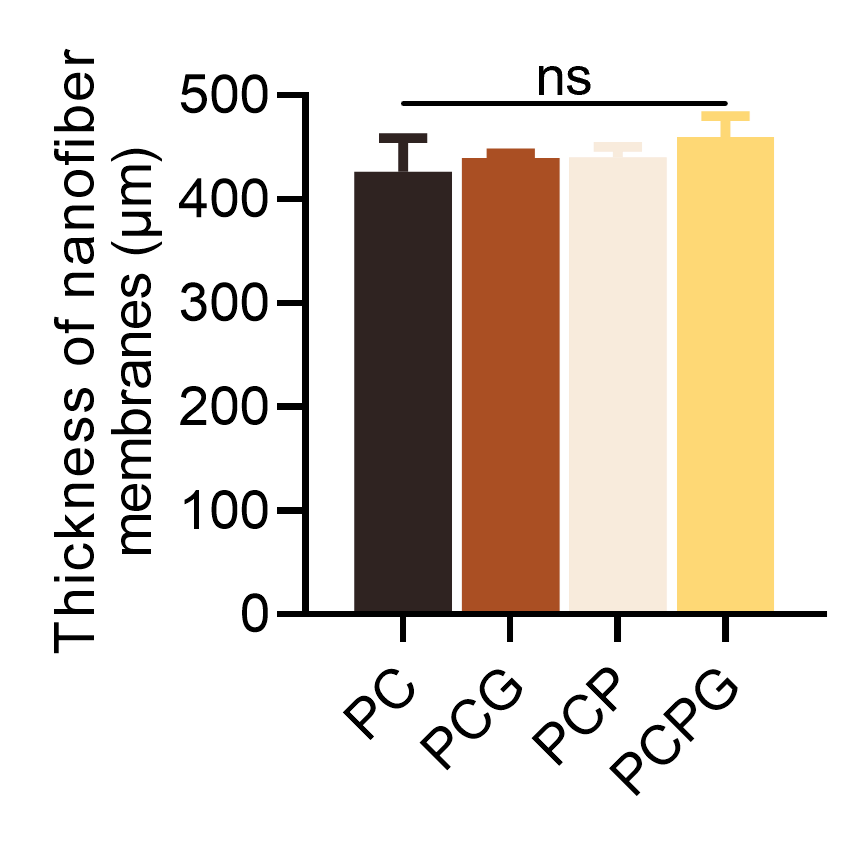


**Figure S2.** Thickness measurement data for PC, PCG, PCP, and PCPG nanofibrous membranes. (n = 3, ns indicates no significant difference)


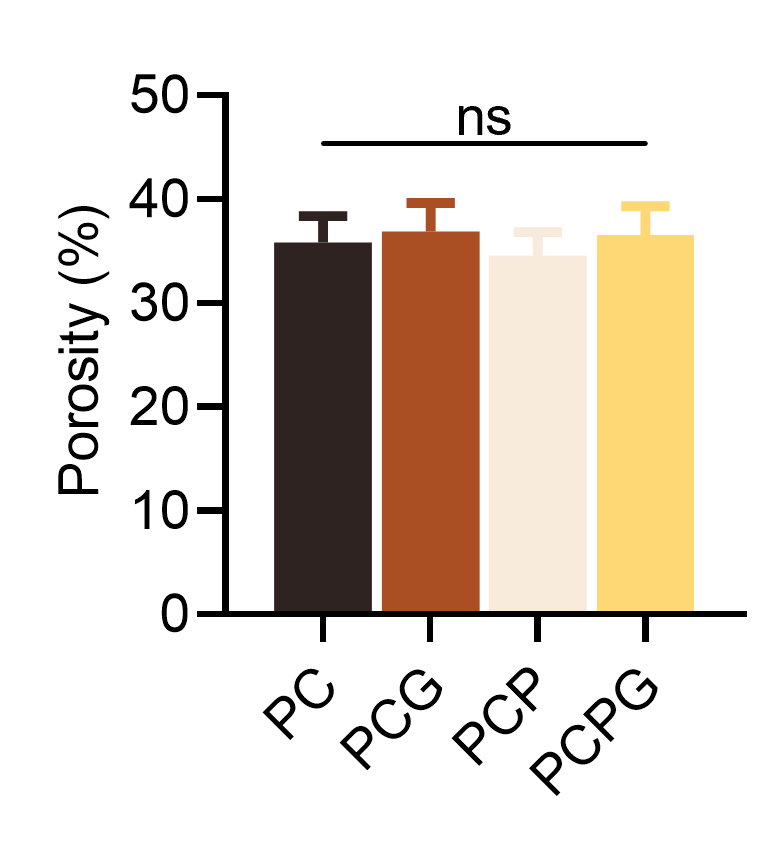


**Figure S3.** Porosity of the PC, PCG, PCP, and PCPG nanofibrous membranes. (n = 3, ns indicates no significant difference)


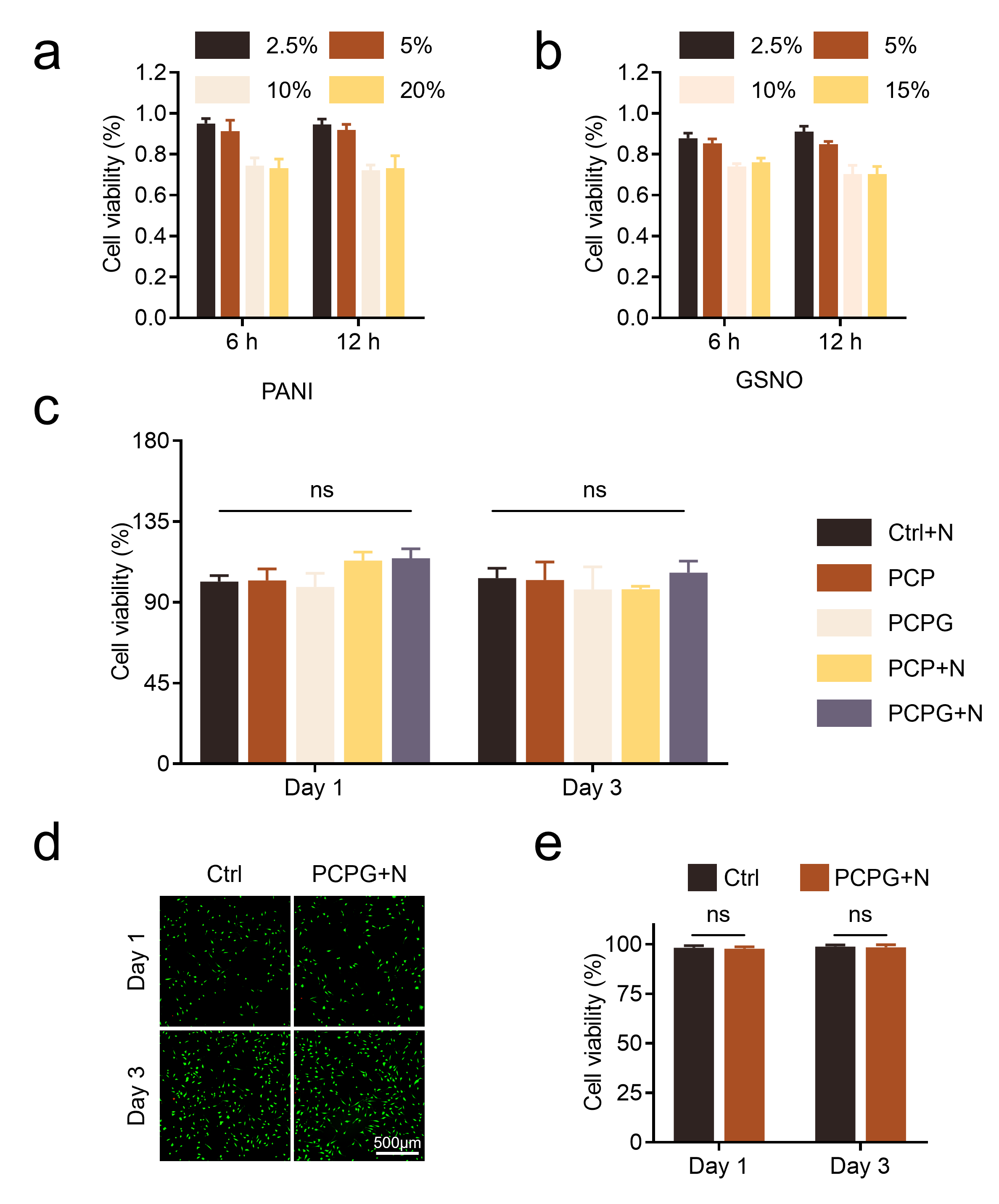


**Figure S4.** Assessment of the biocompatibility of PC-based nanofibrous membranes. (a–b) CCK-8 assays were performed to evaluate the biocompatibility of PC nanofibrous membranes loaded with varying ratios of PANI and GSNO. (c) CCK-8 analysis of the Ctrl+N, PCP, PCPG, PCP+N, and PCPG+N groups. (d) Live/dead staining images of the Ctrl and PCPG+N groups. (e) Quantitative analysis of live/dead staining in the Ctrl and PCPG+N groups. (n = 3, ns indicates no significant difference)


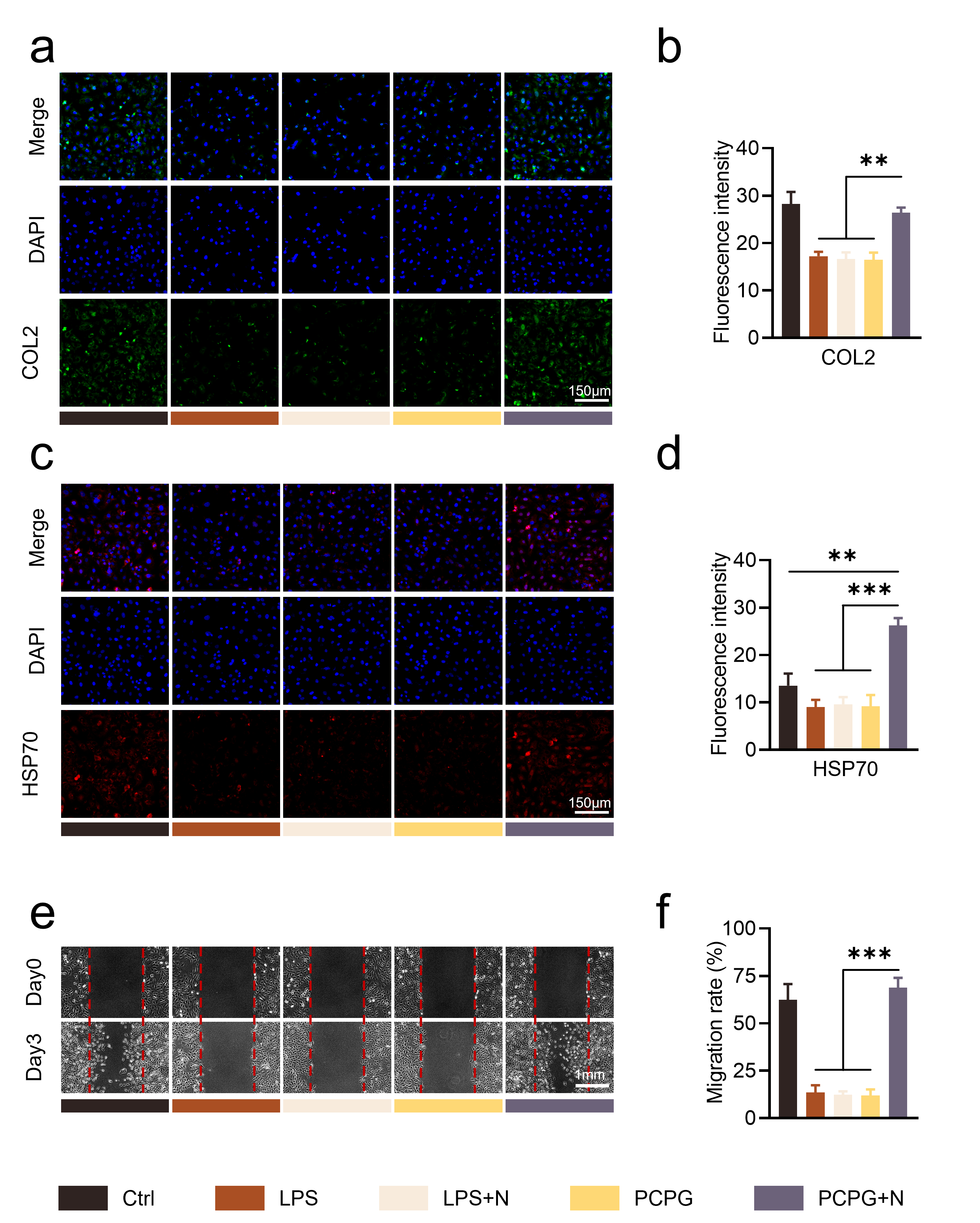


**Figure S5.** Protein expression and migration of AFCs under different treatments (Ctrl, LPS, LPS+N, PCPG, PCPG+N). (a) Immunofluorescence staining image of COL2. (c) Immunofluorescence staining image of HSP70. (b, d) Quantitative analysis of immunofluorescence. (e) The scratch assay outcomes of AFCs on days 0 and 3. (f) Quantitative analysis of the migration rate. (n=3, ∗∗P < 0.01, ∗∗∗P < 0.001)


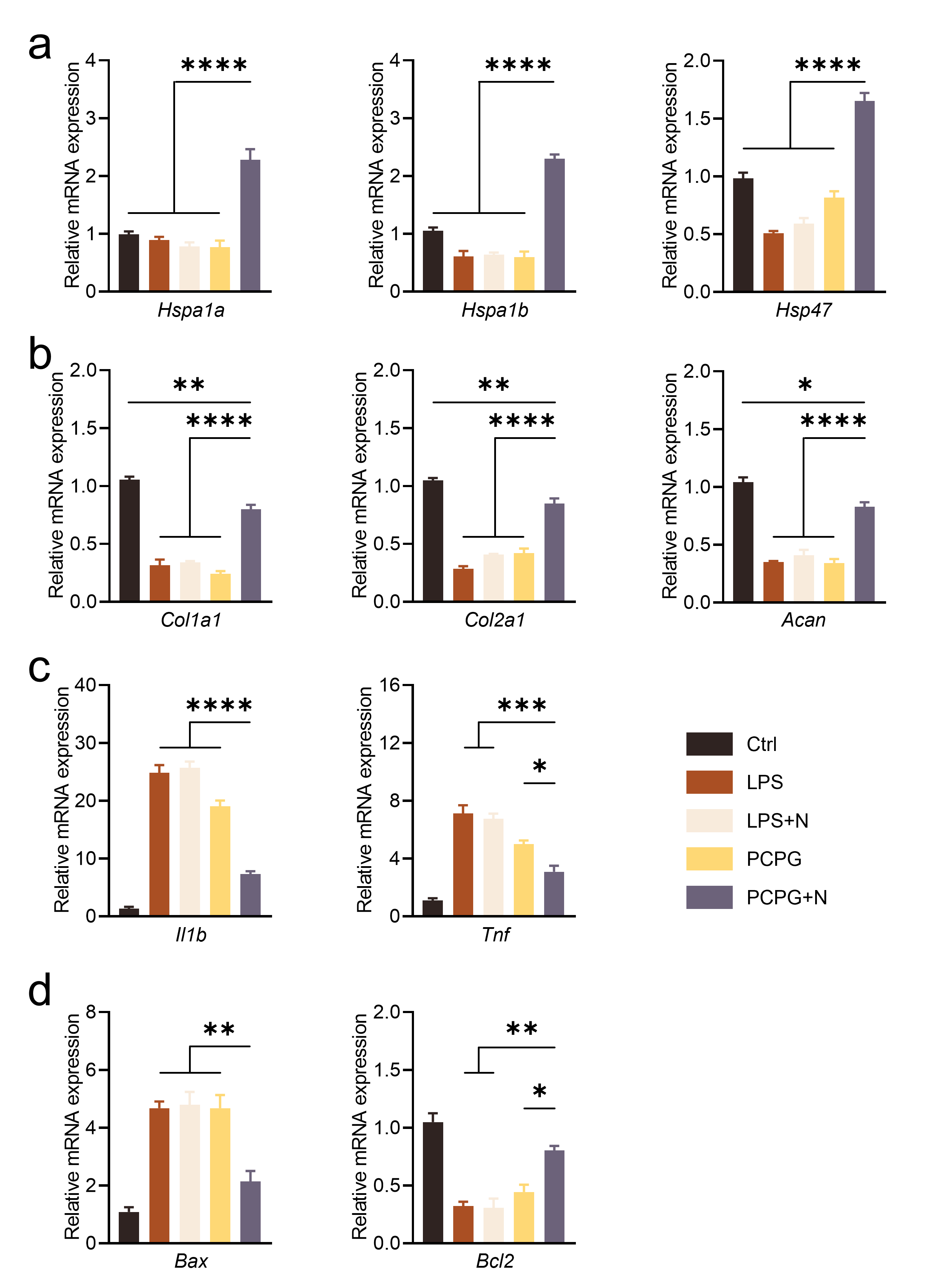


**Figure S6.** Gene expression profiles of AFCs under different treatments (Ctrl, LPS, LPS+N, PCPG, PCPG+N). (a) HSP-related gene expression. (b) Expression of anabolic markers. (c) Inflammatory gene expression. (d) Apoptosis-associated gene expression. (n=3, ∗P < 0.05, ∗∗P < 0.01, ∗∗∗P < 0.001, and ∗∗∗∗P < 0.0001)


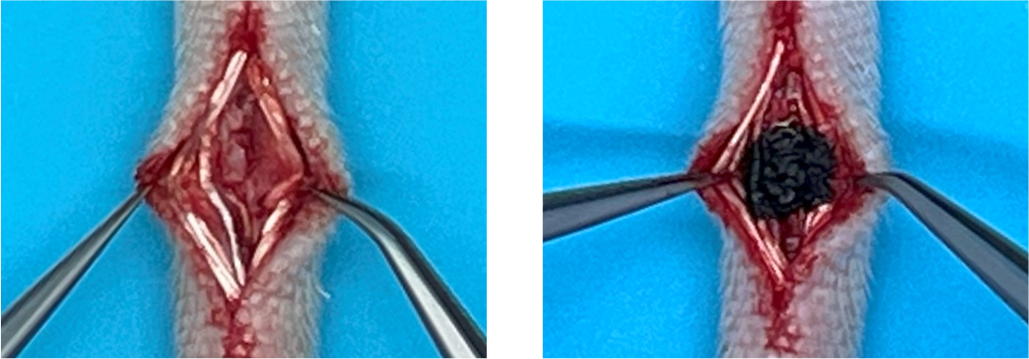


**Figure S7.** Surgical images of PCPG nanofibrous membrane implantation on the annulus fibrosus surface of the rat tail.


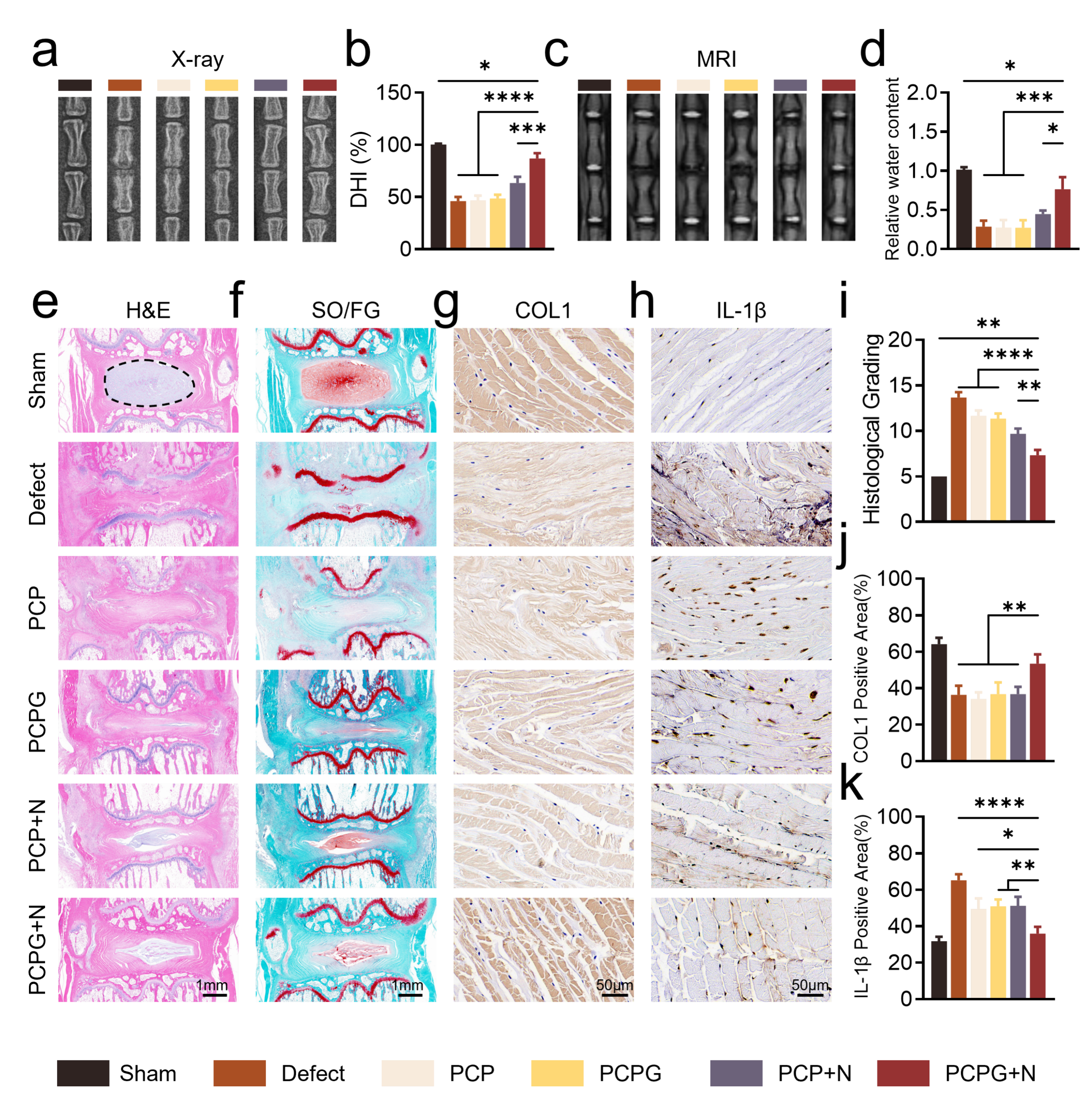


**Figure S8.** In vivo evaluation of IVD regeneration using nanofibrous membranes. (a) X-ray images of rat caudal vertebrae at week 4. (b) DHI quantification. (c) MRI scans of rat caudal vertebrae at week 4. (d) Measurement of relative IVD water content. (e, f) Representative H&E and SO/FG staining of IVD sections from Sham, Defect, PCP, PCPG, PCP+N, and PCPG+N groups at week 4. Black dashed line denotes the AF–NP boundary. (g,h) IHC staining image of COL1 and IL-1β at week 4. (i) Histological grades. (j, k) Quantitative analysis of IHC staining. (n=3, ∗P < 0.05, ∗∗P < 0.01, ∗∗∗P < 0.001, and ∗∗∗∗P < 0.0001)


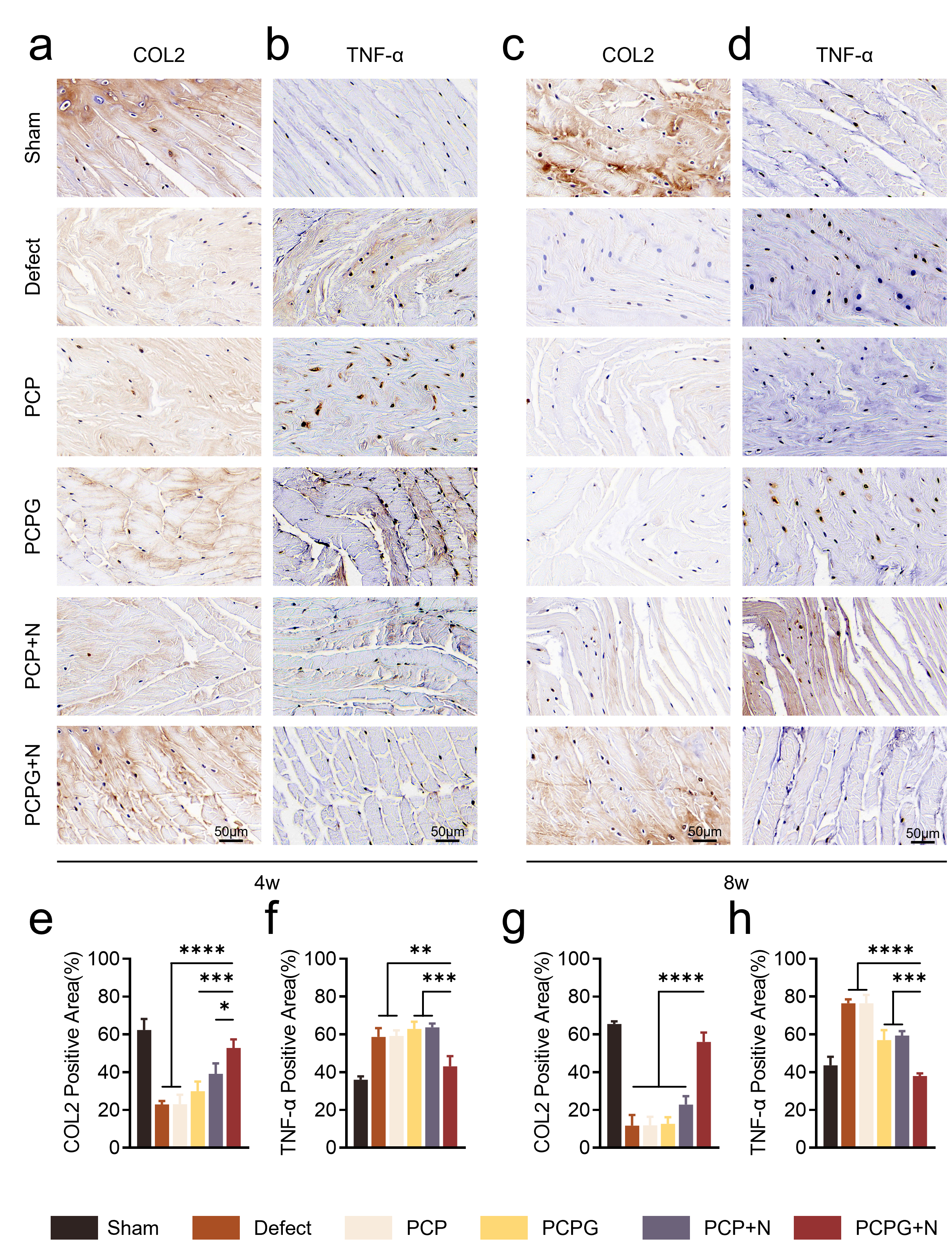


**Figure S9.** In vivo repair of IVD by the nanofibrous membranes. (a-b) IHC staining image of COL2 and TNF-α at week 4. (c-d) IHC staining image of COL2 and TNF-α at week 8. (e-h) Quantitative analysis of IHC staining. (n=3, ∗∗∗P < 0.001, and ∗∗∗∗P < 0.0001)


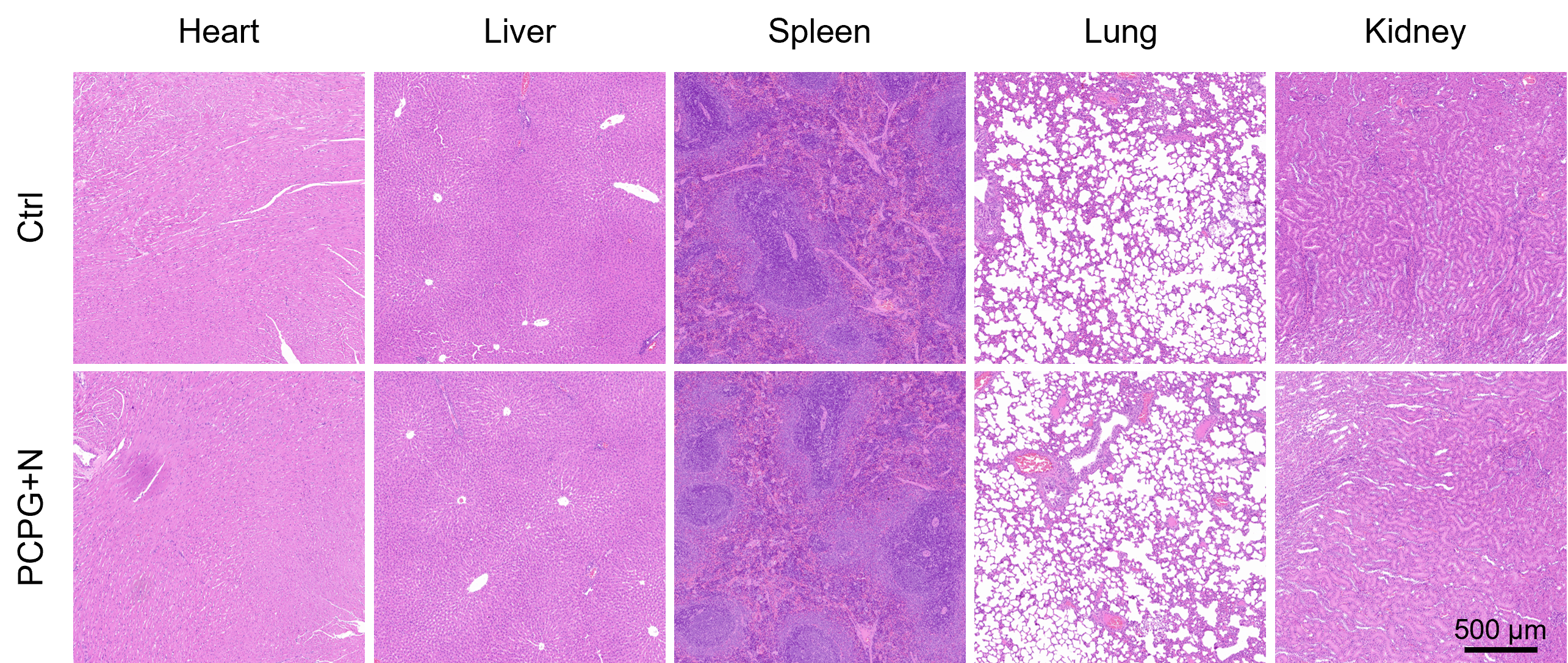


**Figure S10.** H&E staining demonstrated preserved structural integrity of the heart, liver, spleen, lung, and kidney across all groups.


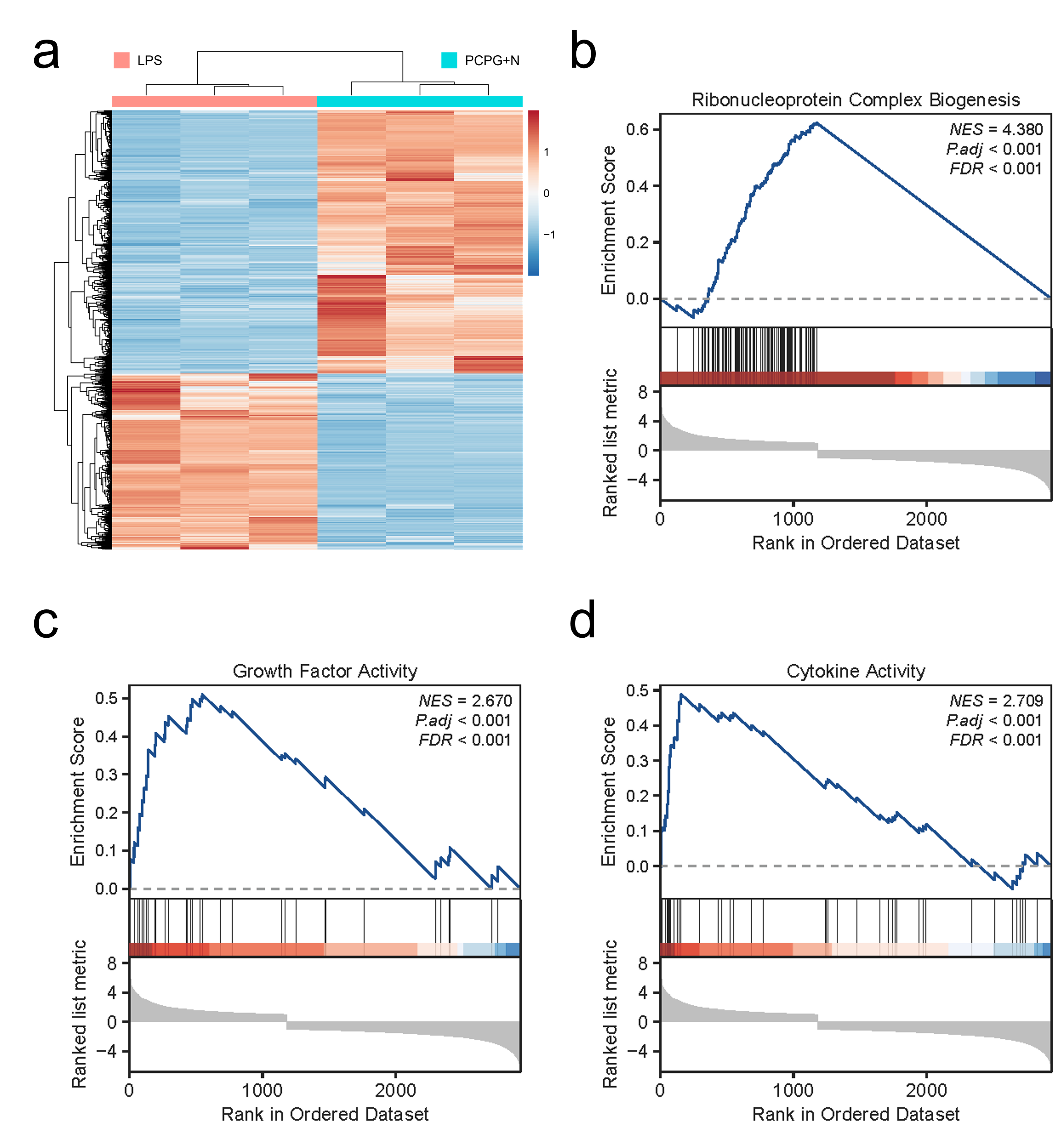


**Figure S11.** Molecular mechanisms underlying synergistic treatment. (a) Heatmap illustrating transcriptomic differences between the LPS and PCPG+N groups. (b–d) GSEA of DEGs associated with key signaling pathways.

**Table S1.** Primer sequences used for qRT-PCR.

| Genes | Forward Primer | Reverse Primer | Id |
| --- | --- | --- | --- |
| *Col1a1* | TGGTCCTGCTGGCAAGAATGG | TCTGTCACCTTGTTCGCCTGTC | NM_053304.1 |
| *Col2a1* | TGGTGGAGCAGCAAGAGCAAG | TGAAGTGGAAGCCGCCGTTC | NM_012929.2 |
| *Acan* | AGTGCTATGCTGGCTGGTTGG | GGTGCTTGGACAGTGGATCAGG | NM_022190.2 |
| *Hspa1a* | CGCTCCAGGTGTGATCTAGG | TTGCAGACCGAACGAAGGAG | NM_031971.2 |
| *Hspa1b* | CGAGGAGGTGGATTAGAGGCT | CAGCAGCCATCAAGAGTCTGT | NM_001329896.2 |
| *Hsp47* | CTGAGTAGAGCCTGCCTGAGA | GAGGACCTGTGAGGGCTTGAA | XM_039107102.2 |
| *Bax* | GTCTCAAGAGGCTTACCAGTAACA | GGGTCCCGAAGTAGGAAAGG | XM_063281064.1 |
| *Bcl2* | TGTGTGGAGAGCGTCAACAG | TAGTTCCACAAAGGCATCCCAG | NM_016993.2 |
| *Il1b* | AGCTTCAGGAAGGCAGTGTCAC | GCTCCACGGGCAAGACATAGG | NM_031512.2 |
| *Tnf* | CCGAGATGTGGAACTGGCAGAG | CCGCCACGAGCAGGAATGAG | NM_012675.3 |
| *GAPDH* | CTGGAGAAACCTGCCAAGTATG | GGTGGAAGAATGGGAGTTGCT | NM_017008.4 |
